# Elucidating the PTK2-Targeted Anti-Hepatocellular Carcinoma Effects of *Euphorbia helioscopia* L. via Integrated Network Pharmacology, Mendelian Randomization, and Experimental Validation

**DOI:** 10.3390/cimb48020213

**Published:** 2026-02-14

**Authors:** Jianhua Zhu, Li Qian, Chuanjun Yuan, Jia Sun, Jie Pan, Ting Liu, Yang Jin, Yongjun Li, Lin Zheng, Chunhua Liu, Yuan Lu

**Affiliations:** 1 State Key Laboratory of Discovery and Utilization of Functional Components in Traditional Chinese Medicine, Engineering Research Center for the Development and Application of Ethnic Medicine and TCM (Ministry of Education), Guizhou Medical University, Guian New Area, Guiyang, 561113, China; jianhuazhu433@gmail.com (J.Z.); sunjia1202@126.com (J.S.); gmupanjie@126.com (J.P.); liuting@gmc.cn (T.L.); jinyang4791@163.com (Y.J.); liyongjun026@126.com (Y.L.); zhengl2020@126.com (L.Z.); 2 School of Pharmacy, Guizhou Medical University, Guian New Area, Guiyang 561113, China; lilly0907momo@163.com (L.Q.); 15585096536@163.com (C.Y.); 3 Guizhou Key Laboratory of Modern Traditional Chinese Medicine Creation, Guian New Area, Guiyang 561113, China

**Keywords:** *Euphorbia helioscopia* L., hepatocellular carcinoma, network pharmacology, mendelian randomization, PTK2/PI3K/AKT signaling pathway, molecular docking

## Abstract

*Euphorbia helioscopia* L. (Zeqi, ZQ) is a traditional Chinese herb used to treat various tumors, but its molecular mechanisms against hepatocellular carcinoma (HCC) remain unclear. This study aims to elucidate the anti-HCC mechanisms of ZQ using chemical profiling, bioinformatics, Mendelian randomization (MR), and experimental validation. A total of 104 compounds were identified from ZQ, with 18 targeting HCC-related proteins. Bioinformatics and MR analyses revealed PTK2 as a core target associated with HCC risk. ZQ significantly suppressed H22 tumor growth in male ICR mice and inhibited PTK2/PI3K/AKT phosphorylation. Molecular docking and dynamics simulations confirmed stable binding between key ZQ compounds and PTK2. These results suggest that ZQ exerts anti-HCC effects through PTK2 inhibition and modulation of the PI3K/AKT pathway, supporting its potential as a multi-targeted therapeutic for HCC.

## 1. Introduction

Liver cancer remains the second leading cause of cancer-related mortality worldwide, with hepatocellular carcinoma (HCC) accounting for approximately 90% of primary liver cancer cases [1]. Despite advances in surgical resection, ablation, transarterial chemoembolization, targeted therapy, immunotherapy, and radiotherapy, the overall prognosis of HCC remains poor, underscoring the urgent need for novel and effective therapeutic strategies [2,3,4,5,6].

Natural products with a history of traditional medicinal use have attracted increasing interest as potential anticancer agents. *Euphorbia helioscopia* L., commonly known as Zeqi (ZQ) in Chinese medicine, is a traditional ethnomedicinal herb first documented in the *Shennong Bencao Jing*. In folk medicine, it has long been used to treat edema, pulmonary diseases, tuberculosis, osteomyelitis, and various tumors [7]. Accumulating pharmacological evidence indicates that ZQ exhibits antitumor activity against multiple malignancies, including HCC [8].

Recent studies suggest that ZQ and its bioactive constituents exert anticancer effects through diverse mechanisms, including inhibition of tumor cell proliferation, induction of apoptosis, modulation of immune responses, and suppression of angiogenesis [9,10,11]. For example, ZQ extracts inhibit hepatitis B virus-related HCC by regulating AKT1 and Caspase-3 signaling [9], while the monomer euphornin induces G2/M cell cycle arrest and apoptosis in cervical cancer cells [12]. These findings support the multi-component and multi-target nature of ZQ. However, its precise anti-HCC mechanisms remain incompletely understood.

Several critical limitations persist in current ZQ-related studies, including insufficient systematic characterization of its active components and targets, limited in vivo validation, and a lack of genetic evidence supporting causal target–disease relationships. Most network pharmacology analyses remain correlation-based and do not provide robust causal inference, highlighting the need for integrative strategies combining multi-omics data and experimental validation.

In this study, we employed an integrated workflow to elucidate the anti-HCC mechanisms of ZQ. The chemical constituents of ZQ ethanol extract were first characterized using Q-Exactive Orbitrap High-Resolution Mass Spectrometry (UHPLC-Q-Exactive Plus Orbitrap HRMS). Network pharmacology was then applied to predict potential therapeutic targets, followed by expression quantitative trait loci (eQTL)-based Mendelian randomization (MR) analysis to infer genetic causal relationships between candidate targets and HCC risk [13,14]. Key targets were further evaluated using public transcriptomic datasets, in vivo validation in an H22 tumor-bearing mouse model, and molecular docking and molecular dynamics (MD) simulations.

By integrating phytochemical profiling, genetic causal inference, experimental validation, and molecular modeling, this study provides novel insights into the pharmacological basis of ZQ and supports its potential development as a multi-target therapeutic agent for HCC.

## 2. Materials and Methods

### 2.1. Plant Materials

*Euphorbia helioscopia* L. (ZQ, Batch No. 20240701) was purchased from Anhui Meikang Pharmaceutical Technology Co., Ltd. (Bozhou, China). and authenticated as the dried aerial parts of *Euphorbia helioscopia* (Family: Euphorbiaceae) by Professor Chunhua Liu from Guizhou Medical University. A voucher specimen has been deposited at the Engineering Research Center for the Development and Application of Ethnic Medicine and TCM (Ministry of Education), Guizhou Medical University, for future reference. Powdered ZQ herbal material was extracted by refluxing with 8 volumes of 70% ethanol solution. After filtration, a portion of the filtrate was passed through a 0.22 μm microporous membrane and analyzed using UHPLC Q-Exactive Plus Orbitrap HRMS (Thermo Fisher Scientific, Waltham, MA, USA). The remaining extract was concentrated under reduced pressure to obtain a paste, and the extraction yield was calculated. For animal experiments, the extract was suspended in 0.5% sodium carboxymethyl cellulose (CMC-Na) for oral gavage.

### 2.2. Chemicals and Reagents

Doxorubicin (DOX, J06248, Meilunbio, Dalian, China) was used as a positive control drug for in vivo experiments. LC-MS-grade formic acid (214911, Thermo, Waltham, MA, USA) and acetonitrile (204197, Thermo, Waltham, MA, USA) were applied for UHPLC-Q-Exactive Plus Orbitrap HRMS analysis.

For protein extraction and Western blotting, key reagents included RIPA lysis buffer (P0013D, Beyotime, Shanghai, China), PMSF (ST505, Beyotime, Shanghai, China), BCA Protein Assay Kit (P0009, Beyotime, Shanghai, China), SDS (S8010-500g, Solarbio, Beijing, China), ammonium persulfate (ST005, Beyotime, Shanghai, China), and acrylamide (800013290, Sinopharm, Shanghai, China). PVDF membranes (IPVH00010, Millipore, Burlington, MA, USA) were used for protein transfer. Primary antibodies used were: anti-GAPDH (ab8245, Abcam, Waltham, MA, USA), anti-PTK2 (12636-1-AP, Proteintech, Wuhan, China), anti-phospho-PTK2 (Tyr397) (#3283, CST, Danvers, MA, USA), anti-PI3K (60225-1-Ig, Proteintech, Wuhan, China), anti-phospho-PI3K (Y607) (AF3241, Affinity Biosciences, Changzhou, China), anti-AKT (10176-2-AP, Proteintech, Wuhan, China), and anti-phospho-AKT (Ser473) (#4060, CST, Danvers, MA, USA). The corresponding HRP-conjugated secondary antibodies were purchased from Jackson ImmunoResearch (West Grove, PA, USA). Protein bands were visualized using an enhanced chemiluminescence (ECL) substrate (BL520A, Biosharp, Hefei, China).

### 2.3. Main Instruments

Vanquish UPLC system, Q Exactive Plus high-resolution mass spectrometer, and the proprietary OTCML local TCM compound MS database (Thermo Fisher Scientific, Waltham, MA, USA); Allegra X-30R Centrifuge (Beckman Coulter, Brea, CA, USA); Electrophoresis apparatus (DYY-6C, Liuyi, Beijing, China); Microplate reader (EnSpire, PerkinElmer, Waltham, MA, USA); Imaging system (LEICA DM750, Leica Instruments Shanghai Co., Ltd., Shanghai, China).

### 2.4. UHPLC Q-Exactive Plus Orbitrap HRMS Analysis of ZQ Components

#### 2.4.1. UHPLC-HRMS Conditions

Qualitative analysis of chemical constituents in ZQ was performed using UHPLC-Q Exactive Plus Orbitrap HRMS. The chromatographic separation was carried out on a Hypersil Gold column (100 mm × 2.1 mm, 1.9 μm). The mobile phase consisted of A: 0.1% formic acid in water, and B: 0.1% formic acid in acetonitrile, with the following gradient elution: 0–1 min: 98% A; 1–2 min: 98% → 95% A; 2–20 min: 95% → 34% A; 20–26 min: 34% → 2% A; 26–27 min: 2% A; 27–27.5 min: 2% → 98% A; 27.5–30 min: 98% A. Flow rate: 0.3 mL/min; injection volume: 1 μL; column temperature: 40 °C.

Mass spectrometry was performed with an ESI source in both positive and negative ion modes. Source parameters: capillary voltage 3.5 kV (positive mode), 2.5 kV (negative mode); ion source temperature: 110 °C; sheath gas: 35 arb; auxiliary gas: 10 arb; capillary temperature: 320 °C; probe heater: 350 °C; S-lens RF level: 50.0; normalized collision energy values: 20/40/60 eV; acquisition mode: Full MS + data-dependent MS/MS (ddMS^2^); Full MS resolution: 70,000; MS/MS resolution: 17,500.

#### 2.4.2. GNPS Molecular Networking

The raw MS/MS data were converted to mzXML format using MSConvert. The converted data were uploaded to the GNPS platform (https://gnps.ucsd.edu/, accessed on 1 May 2025), and molecular networking was constructed using its online workflow. Clusters were generated based on spectral similarity scores above 0.7. The resulting molecular networks were visualized using Cytoscape (v3.10.2).

#### 2.4.3. Data Analysis

Raw spectra were imported into Compound Discoverer 3.0. Parameters were set as follows: retention time (RT) tolerance: 0.5 min; *m*/*z* error: 5 ppm; peak detection threshold: *m*/*z* 1.5. Information such as RT, *m*/*z*, adduct type, and match score were obtained. MS/MS fragments were extracted using Thermo Xcalibur 4.1. Compounds were identified by matching calculated and theoretical molecular weights (with a mass accuracy < 5 ppm), and confirmed with *m*/*z* Cloud, PubChem (https://pubchem.ncbi.nlm.nih.gov, accessed on 1 May 2025) [15], and published literature.

### 2.5. Network Pharmacology

Based on chemical profiling results, the TCMSP platform (https://www.tcmsp-e.com, accessed on 3 May 2025) was used to screen for active compounds in ZQ with oral bioavailability ≥ 30% and drug-likeness ≥ 0.18 [16]. Potential targets were retrieved via PubChem and SwissTargetPrediction [17] (http://www.swisstargetprediction.ch/index.php, accessed on 3 May 2025) (probability > 10), and gene names were standardized using UniProt (https://www.uniprot.org, accessed on 3 May 2025) [18]. HCC-related targets were obtained from GeneCards (https://www.genecards.org, accessed on 3 May 2025) [19] and OMIM databases (https://omim.org, accessed on 3 May 2025) [20]. The overlapping targets were uploaded to STRING (https://string-db.org, accessed on 3 May 2025) [21] for protein–protein interaction (PPI) network construction. Core targets were identified using the CytoHubba and molecular complex detection (MCODE) plugins in Cytoscape (v3.10.2). Gene Ontology (GO) and Kyoto Encyclopedia of Genes and Genomes (KEGG) enrichment analyses were performed using DAVID (https://davidbioinformatics.nih.gov, accessed on 3 May 2025) [22], and results were visualized in R (v4.4.2). A comprehensive “herb–compound–core target–key pathway–disease” network was then constructed [23,24].

### 2.6. Data Sources and MR Analysis

Whole blood cis-eQTL data were obtained from the eQTLGen consortium (https://www.eqtlgen.org, accessed on 5 May 2025) (n = 31,684; 16,987 genes), primarily from healthy individuals of European ancestry [25]. To enhance statistical power and minimize weak instrument bias inherent to currently available liver-specific eQTL datasets, we leveraged large-scale whole-blood cis-eQTL data. Importantly, extensive meta-analyses have demonstrated that cis-eQTL effects, particularly those regulating ubiquitously expressed genes, are largely shared across tissues and show high concordance in allelic direction between blood and solid organs, including the liver [26,27]. To further ensure regulatory fidelity, we prioritized single nucleotide polymorphisms (SNPs) with stringent *F*-statistics (>20) and restricted our analysis to cis-acting variants of ubiquitously expressed genes. After integrating allele frequency data, we calculated Effect Allele Frequency, beta, and standard errors (from Z-scores and sample sizes). Genome-wide significant SNPs (*p* < 5 × 10^−8^) were pruned for linkage disequilibrium (*r*^2^ < 0.001, 10 Mb window), and only those with *F*-statistics > 20 were retained and grouped by gene.

GWAS summary statistics for HCC were sourced from the GWAS Catalog (https://www.ebi.ac.uk/gwas, accessed on 3 May 2025) (123 cases, 456,225 controls, 11.8 M SNPs), all of European ancestry [28]. These publicly available data required no ethical approval.

To explore the causal impact of eQTLs associated with the 11 previously identified key genes on HCC, we conducted two-sample MR analysis using the TwoSampleMR R package (v 0.5.8) [29]. Exposure and outcome datasets were harmonized to align alleles and correct strand mismatches. Only SNPs with *F* > 10 and *p* < 0.05 for the exposure were used as instruments. Causal effects were estimated via IVW, with odds ratios and 95% CIs reported. Horizontal pleiotropy was assessed using MR–Egger intercept, and heterogeneity via Cochran’s Q. The MR-PRESSO global test (1000 permutations) was used to detect pleiotropic outliers. Sensitivity analyses included single SNP effects, leave-one-out testing, and funnel plot evaluation. Analyses were conducted in R (v4.4.2).

### 2.7. Differential Expression Profiling and ROC Curve Analysis of PTK2 in HCC

The core target PTK2 was identified by intersecting key targets obtained from network pharmacology and MR analyses. The differential expression of PTK2 in HCC was examined using the Integrative Molecular Database of Hepatocellular Carcinoma (HCCDB) (http://lifeome.net:809/#/home, accessed on 3 May 2025) [30] and Human Protein Atlas (HPA) (https://www.proteinatlas.org, accessed on 3 May 2025) [31].

The Cancer Genome Atlas (TCGA) database (https://portal.gdc.cancer.gov, accessed on 10 May 2025), established by the National Cancer Institute, contains comprehensive genomic, transcriptomic, proteomic, and methylation data from approximately 20,000 primary cancer cases. To evaluate the significance of the PTK2 gene, we retrieved transcriptomic data and corresponding clinical information for liver tissues from 369 patients with liver hepatocellular carcinoma (LIHC) and 50 normal individuals [32]. Differential expression analysis was performed using the “DESeq2” package in R, and the results were visualized accordingly. We compared the expression levels of PTK2 between normal and tumor samples. For consistency throughout the manuscript, LIHC is uniformly referred to as HCC. Subsequently, ROC curve analysis was conducted to assess the diagnostic sensitivity (true positive rate) and specificity (true negative rate) of PTK2 for HCC. The area under the curve (*AUC*) was calculated using the “pROC” package in R to predict the diagnostic value of this key gene based on ROC curve analysis.

### 2.8. Animal Treatment

#### 2.8.1. Animals and Housing Conditions

Twenty-four male ICR mice (4 weeks old, 20 ± 2 g) were purchased from SPF (Beijing) Biotechnology Co., Ltd. (License No. SCXK [Jing] 2024-0001, Beijing, China) and housed in a specific pathogen-free (SPF) facility. The animals were maintained under standard conditions, including a 12-h light/dark cycle, a temperature range of 18–25 °C, and a relative humidity of 50–70%. They had ad libitum access to food and water. All experimental procedures were performed in strict compliance with the ARRIVE (https://arriveguidelines.org, accessed on 10 May 2025) guidelines and the Guidelines for the Care and Use of Laboratory Animals. The protocol received ethical approval from the Animal Ethics Committee of Guizhou Medical University (Ethical approval No. 2503375). Animals were sacrificed upon reaching ethical endpoints that include, but are not limited to, breathing difficulties,  ≥20% body weight loss or 2000 mm^3^ tumor volume.

#### 2.8.2. Cell Culture and Tumor Induction

The murine HCC cell line H22 (Cat# 20200929) was obtained from Wuhan Procell Life Science & Technology Co., Ltd, (Wuhan, China). Cells were cultured in RPMI-1640 medium supplemented with 10% fetal bovine serum and 1% penicillin–streptomycin at 37 °C in a 5% CO_2_ incubator. Subculturing was performed every 2–3 days. For tumor induction, H22 cells in the logarithmic growth phase were harvested and resuspended in phosphate-buffered saline (PBS) at a concentration of 1 × 10^7^ cells/mL, and 0.2 mL of the suspension was injected intraperitoneally into the mice. Five days later, ascites formed, and the ascitic fluid was collected. The cell density was adjusted to 1 × 10^7^ cells/mL under sterile conditions, and 0.2 mL of the suspension was injected into the right axillary region of the mice. After approximately 5 days, a small lump appeared at the injection site, confirming the successful establishment of the tumor model.

#### 2.8.3. Experimental Design and Treatment

After tumor establishment, a total of 24 mice were randomly divided into four groups (*n* = 6 per group): Control Group: 0.25 mL physiological saline daily via oral gavage. DOX Group: DOX at 3 mg/kg every other day via tail vein injection. ZQ Low-Dose Group (ZQL): ZQ extract at 232 mg/kg (0.91 g/kg ZQ) daily via oral gavage. ZQ High-Dose Group (ZQH): ZQ extract at 928.2 mg/kg (3.64 g/kg ZQ) daily via oral gavage. Treatment continued for 14 consecutive days.

#### 2.8.4. Assessment of Antitumor Efficacy and Histopathological Evaluation

During the treatment period, the general condition of the mice was carefully monitored, and their body weight was recorded on a daily basis. Tumor dimensions were measured every two days using a digital caliper. Tumor volume was calculated using the formula: *V* = (*a* × *b*^2^) × 0.5, where a represents the tumor’s longest diameter (length) and b the shortest (width). Based on these measurements, tumor growth curves were generated for each group, Treatments and measurements followed a consistent sequence.

At the end of the treatment period, all animals were humanely sacrificed via CO_2_ inhalation anesthesia followed by cervical dislocation. Tumors were harvested, weighed, and the tumor inhibition rate (TIR) was calculated using the following equation:

TIR (%) = [(C − T)/C] × 100%,

where C denotes the average tumor weight of the model control group, and T refers to that of the treatment group.

For histopathological analysis, three representative tumor samples from each group were fixed and subjected to Hematoxylin and Eosin (H&E) staining. The remaining tumor tissues were snap-frozen and preserved at −80 °C for subsequent analysis of protein expression levels using Western blotting.

### 2.9. Western Blot Analysis

Frozen tumor tissue samples were homogenized in ice-cold lysis buffer containing protease inhibitors to prepare total protein solutions. Protein concentrations in the tissue homogenates were determined using the BCA protein assay kit. Subsequently, proteins were separated by SDS-PAGE and transferred to a PVDF membrane, which was then blocked with 5% non-fat dry milk. The membrane was washed five times with TBST, followed by incubation with primary antibodies at 4 °C. The primary antibodies were diluted as follows: GAPDH (1:5000), PTK2 (1:20,000), p-PTK2 (1:1000), PI3K (1:5000), p-PI3K (1:1000), AKT (1:2000), and p-AKT (1:2000). After primary antibody incubation, the membrane was incubated with HRP-conjugated secondary antibodies at room temperature for 1 h. Finally, protein bands were visualized using an ECL detection kit, and protein expression levels were analyzed with a gel imaging system and ImageJ software (v1.54p).

### 2.10. Molecular Docking and MD Simulations

To evaluate the interactions between the core target PTK2 and the 18 active compounds of ZQ identified via network pharmacology, molecular docking analysis was performed. Additionally, the known PTK2 inhibitor Defactinib was included in the docking analysis as a comparative control to contextualize the binding affinities of the ZQ compounds. The 3D structures of the active compounds and Defactinib were retrieved in mol2 format from the PubChem database, while the target protein structures were obtained from RCSB Protein Data Bank (PDB) (https://www.rcsb.org, accessed on 15 May 2025). Protein structures were selected based on the following criteria: human origin, high-resolution crystallographic data, and the presence of native ligands structurally similar to the candidate compounds.

The selected structures were imported into PyMOL (v2.3.0) and AutoDockTools (v1.5.7) for preprocessing. This included removal of water molecules and ligands, addition of hydrogen atoms, optimization of amino acid residues, and charge assignment. All rotatable bonds in the ligands were set to be flexible. Structural files were converted into PDBQT format using Open Babel GUI (v3.1.1). Semi-flexible molecular docking was then conducted using AutoDockTools, and the results were visualized in PyMOL. Docking complexes with binding free energies lower than −5.0 kcal/mol were selected for further MD simulations [33].

MD simulations of the PTK2 protein in complex with Euphornin F, Helioscopinolide C, Helioscopinolide B, Kaempferol, Beta-Sitosterol, Quercetin, and Euphoscopin E were performed using GROMACS [34]. Protein structures were first repaired using Swiss-PdbViewer 4.10, and ligand structures were saved in mol2 format via PyMOL. Ligand topology files were generated using Sobtop 1.0 (dev3.1). Subsequent MD simulations in GROMACS included preparation of protein and topology files, definition of the simulation box, solvation with water molecules, addition of counter-ions, energy minimization, NVT equilibration, and execution of the production simulation.

After the simulation, the stability of the binding interaction was evaluated using GROMACS (version 2025.1). Specifically, the root mean square deviation (RMSD) of the protein–ligand complex was calculated to assess its overall conformational stability and convergence. In addition, the root mean square fluctuation (RMSF) was computed to examine local flexibility and to identify regions, such as binding sites or loops, that became more rigid or more dynamic upon ligand binding [35].

### 2.11. Statistical Analysis

Data are expressed as mean ± standard error of the mean (SEM). Statistical analyses were performed using GraphPad Prism (GraphPad Software, v9.0, San Diego, CA, USA). Before performing parametric tests, the normality of data distribution was assessed using the Shapiro–Wilk test, and the homogeneity of variances was confirmed using Levene’s test. One-way ANOVA followed by Tukey’s post hoc test was used to compare multiple groups. A value of *p* < 0.05 was considered statistically significant.

## 3. Results

### 3.1. Chemical Composition Analysis of ZQ

**Figure 1:**
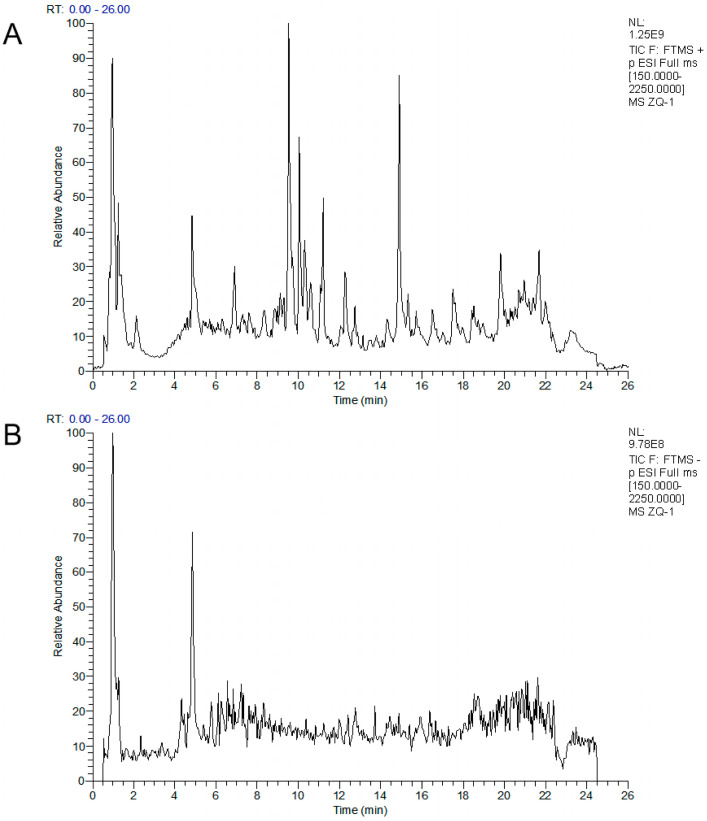
TICs of the ethanol extract of ZQ under different ionization modes. (**A**) Positive-ion mode (ESI^+^). (**B**) Negative-ion mode (ESI^−^). Chromatograms were acquired in full-scan mode (*m*/*z* 150–2250) using an FTMS instrument.

The extraction yield of the ZQ extract was 25.5%, with 255 g of extract obtained from 1 kg of ZQ raw material. After analysis using UHPLC Q-Exactive Plus Orbitrap HRMS, the compound information generated from the mass spectrum was processed on the GNPS platform. The Feature-Based Molecular Network (FBMN) was constructed based on the cosine similarity between MS/MS spectra, visualizing the relationships between similar molecules. The molecular network was visualized using Cytoscape (v3.10.2), as shown in Figure S1. Qualitative analysis of the components was carried out based on the RT, precise relative molecular mass, and secondary mass spectrum fragment information provided by the mass spectrum. The total ion chromatograms (TICs) under positive and negative ion scanning modes are shown in Figure 1. A total of 104 chemical components were identified from the ZQ extract, including 31 flavonoids, 20 organic acids, 9 terpenoids, 8 amino acids, 2 alkaloids, 6 sugars, and 28 other classes. Detailed information on the identified compounds is provided in Table S1.

### 3.2. Network Pharmacology-Based Prediction of the Effects of ZQ in HCC

**Figure 2:**
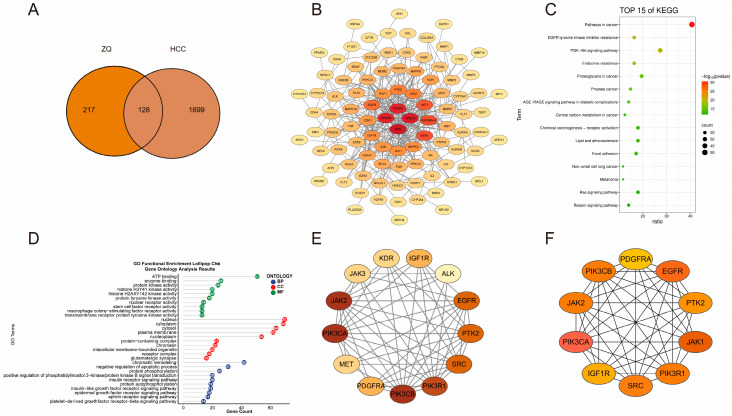
Network pharmacology analysis of ZQ against HCC. (**A**) Venn diagram showing the overlap between ZQ-related targets and HCC-related genes, identifying 128 common targets. (**B**) PPI network of the 128 overlapping targets. (**C**) Top five enriched KEGG pathways. (**D**) Top five enriched terms in the GO categories of BP, MF, and CC. (**E**) Key functional modules identified from the PPI network using the MCODE algorithm. (**F**) Top 10 hub genes identified by the Maximal Clique Centrality algorithm in the CytoHubba plugin.

A total of 18 potential bioactive compounds of ZQ were screened using the TCMSP platform (Table S2) and their corresponding 345 predicted targets were collected from the TCMSP database. HCC-related targets were retrieved from the GeneCards and OMIM databases using the keyword “Hepatocellular carcinoma”, resulting in 1827 non-redundant disease-associated targets. Venn diagram analysis identified 128 overlapping targets between the active components of ZQ and HCC (Figure 2A).

These 128 common targets were imported into the STRING database to construct a PPI network. Using a high confidence threshold (score ≥ 0.9), disconnected nodes were removed, resulting in a network comprising 98 nodes and 315 edges (Figure 2B).

KEGG pathway enrichment analysis identified 263 significantly enriched pathways. The top five included EGFR tyrosine kinase inhibitor resistance, endocrine resistance, PI3K-Akt signaling pathway, prostate cancer, and proteoglycans in cancer (Figure 2C).

GO enrichment analysis was performed on the 128 overlapping targets (Figure 2D). In the Biological Process (BP) category, the enriched terms included gland development, positive regulation of kinase activity, positive regulation of transferase activity, protein autophosphorylation, and response to oxidative stress. In the cellular component (CC) category, the targets were mainly located in the transferase complex, vesicle lumen, PI3K complex class IA and class I, and nuclear chromosome. In the molecular function (MF) category, the enriched functions involved ligand-activated transcription factor activity, nuclear receptor activity, protein serine/threonine kinase activity, protein serine kinase activity, and transmembrane receptor protein tyrosine kinase activity.

Key genes were further identified using the CytoHubba and MCODE plugins in Cytoscape. CytoHubba analysis yielded 13 hub genes: KDR, IGF1R, ALK, EGFR, PTK2, SRC, PIK3R1, PIK3CB, PDGFRA, MET, PI3KCA, JAK2, and JAK3 (Figure 2E). MCODE analysis identified ten core genes, including PDGFRA, EGFR, PTK2, JAK1, PIK3R1, SRC, IGF1R, PIK3CA, JAK2, and PIK3CB (Figure 2F).

**Figure 3:**
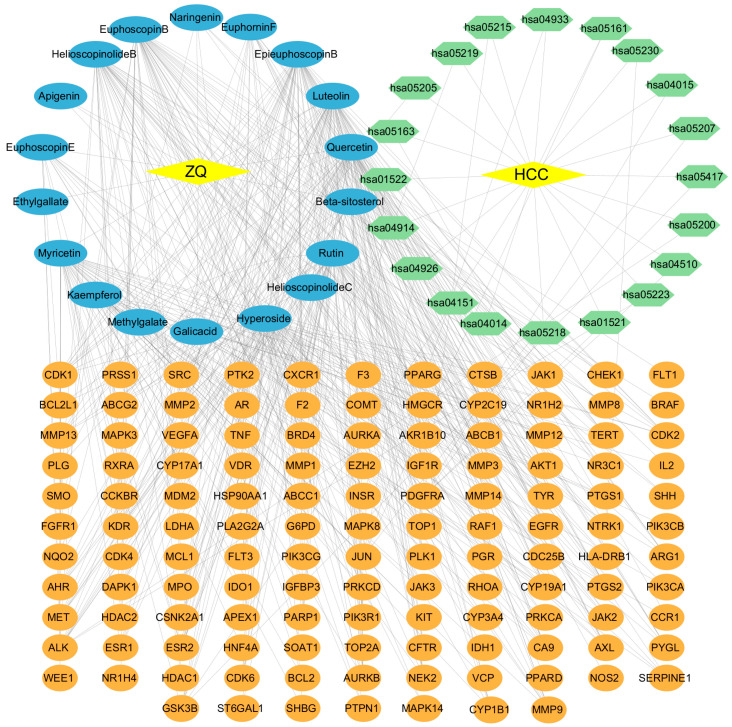
Integrated “herb–compound–core target–key pathway–disease” network of ZQ against HCC. This network illustrates the multi-component, multi-target, and multi-pathway interactions between ZQ (yellow rhombus), its 18 active compounds (blue ellipses), 128 predicted HCC-related targets (orange circles), 20 significantly enriched KEGG pathways (green hexagons), and the associated disease (yellow rhombus, HCC). The visualization highlights the potential pharmacological mechanisms of ZQ in treating HCC through systems-level regulation.

Finally, the 18 potential active compounds, 128 overlapping targets, and key KEGG pathways were integrated using Cytoscape (v3.10.2) to construct a comprehensive “herb–compound–core target–key pathway–disease” network, consisting of 169 nodes and 503 edges (Figure 3).

### 3.3. MR Analysis Results

**Figure 4:**
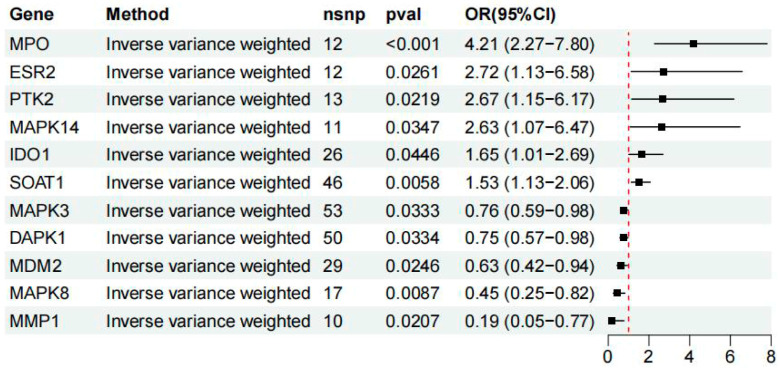
Summary of MR results for candidate genes associated with HCC. Forest plot showing the *ORs* and 95% CIs for 11 genes significantly associated with HCC risk based on IVW MR analysis. The number of instrumental SNPs used for each gene is indicated (nsnp). Genes with *OR* > 1 were positively associated with HCC risk, while those with *OR* < 1 showed potential protective effects.

Based on the IVW method, MR analysis (Figure 4) identified 11 candidate targets (MPO, ESR2, PTK2, MAPK14, IDO1, SOAT1, MAPK3, DAPK1, MDM2, MAPK8, and MMP1) that showed significant causal associations with the risk of HCC.

Among these, six targets, MPO, ESR2, PTK2, MAPK14, IDO1, and SOAT1, were positively associated with increased HCC risk (*OR* > 1, *p* < 0.05), indicating a potential promotive effect. Conversely, MAPK3, DAPK1, MDM2, MAPK8, and MMP1 were negatively associated with HCC risk (*OR* < 1, *p* < 0.05), suggesting a protective effect. All significant associations passed the pleiotropy test (e.g., MR–Egger intercept, *p* > 0.05), and the instrumental variables showed adequate strength, with 10–53 SNPs per target and *F*-statistics > 20.

### 3.4. Identification of Core Target of ZQ in Treating HCC

**Figure 5:**
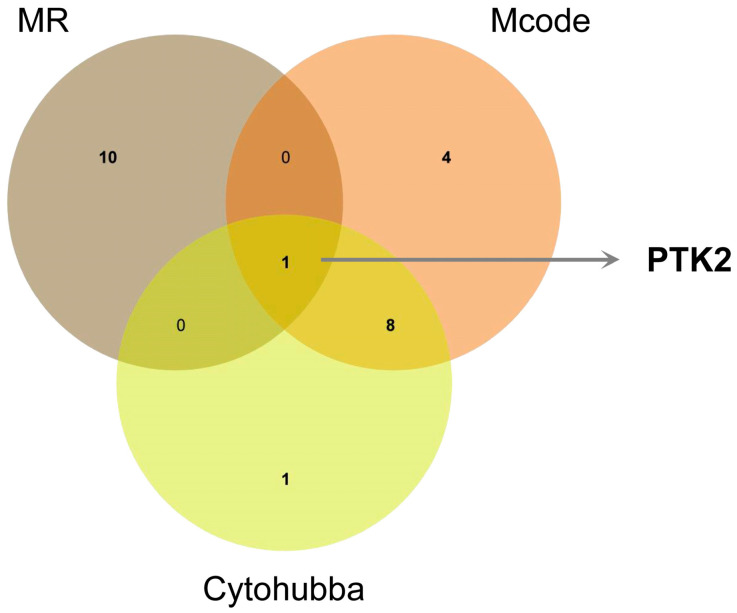
Identification of the core target PTK2 by integrative analysis. Venn diagram illustrating the intersection of candidate genes identified from MR analysis, MCODE clustering, and CytoHubba topological ranking. PTK2 was the only gene shared across all three methods and was thus selected as the core target.

To determine the core therapeutic target of ZQ against HCC, we integrated the results from network pharmacology and MR analysis. By intersecting the core targets identified from two network pharmacology approaches with those from MR analysis, PTK2 was identified as the common key target (Figure 5).

### 3.5. MR Analysis of PTK2

**Figure 6:**
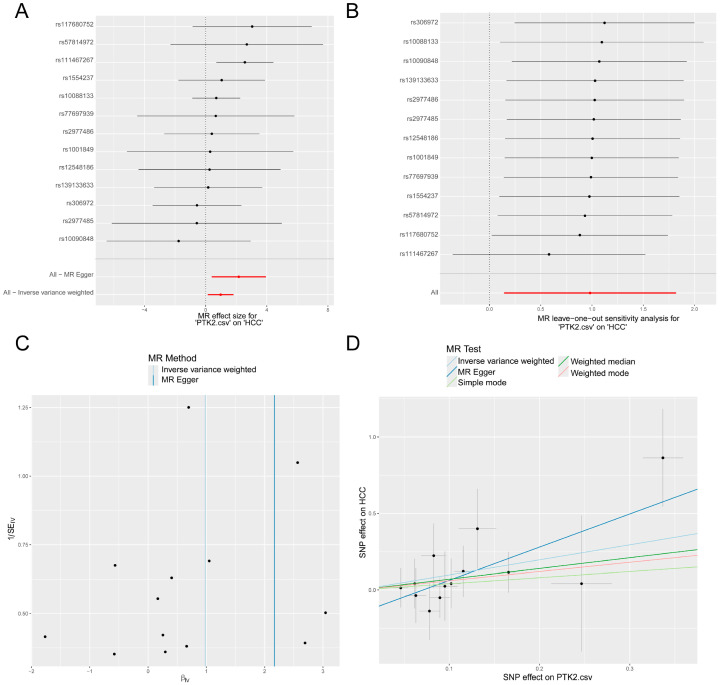
MR analysis supports a causal effect of PTK2 on HCC risk. (**A**) Forest plot illustrating the causal effect estimates of PTK2 on HCC based on individual SNPs used as instrumental variables. Both IVW and MR–Egger methods indicate that increased genetically predicted PTK2 expression is associated with a higher risk of HCC. (**B**) Leave-one-out sensitivity analysis. The consistent causal estimates upon sequential exclusion of each SNP demonstrate the robustness of the association and confirm that it is not driven by any single instrumental variable. (**C**) Funnel plot assessing the potential for directional pleiotropy and heterogeneity. The symmetrical distribution of the SNPs suggests the absence of significant horizontal pleiotropy. (**D**) Scatter plot showing the SNP effects on PTK2 expression versus SNP effects on HCC risk. The positive slopes derived from multiple MR methods (IVW, MR–Egger, weighted median, weighted mode, and simple mode) collectively reinforce a positive causal relationship.

As shown in the forest plot (Figure 6A), MR analysis suggested a positive causal effect of PTK2 on HCC, indicating that increased expression of PTK2 may elevate the risk of developing HCC. To evaluate the robustness of this association, a leave-one-out sensitivity analysis was performed by sequentially removing individual SNPs. The results remained consistent across all iterations (Figure 6B), suggesting that the observed causal relationship was not driven by a single instrumental variable. Funnel plot analysis demonstrated a roughly symmetrical distribution (Figure 6C), implying no substantial heterogeneity or horizontal pleiotropy. In the scatter plot (Figure 6D), each dot represents a selected instrumental SNP, with the x-axis denoting the SNP’s effect on the exposure (PTK2) and the y-axis on the outcome (HCC). The slopes of the regression lines from five different MR methods were all positive, reinforcing the hypothesis of a positive causal relationship between PTK2 and HCC. These MR findings, which identified PTK2 as a core causal gene linked to HCC risk, directly guided our selection of PTK2 for further experimental validation through in vivo studies. Collectively, these findings provide robust evidence for a significant and stable causal association between PTK2 and HCC.

### 3.6. Multi-Omics Evidence for Elevated PTK2 Expression and Its Diagnostic Potential in HCC

**Figure 7:**
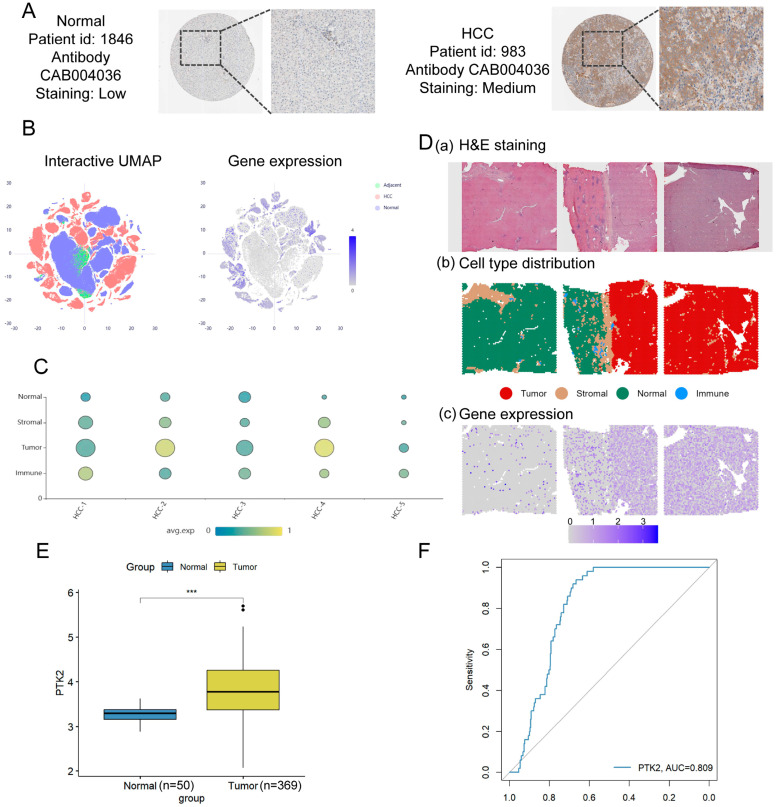
Multi-omics analyses reveal consistent upregulation of PTK2 in HCC and its diagnostic potential. (**A**) Representative IHC images from the HPA show stronger PTK2 protein expression in HCC tissue (Patient ID: 983; moderate intensity) compared to normal liver tissue (Patient ID: 1846; low intensity). (**B**) UMAP plots visualizing PTK2 gene expression across different tissue types. Left: cells colored by their tissue type (Normal, Adjacent, Tumor). Right: projection of PTK2 expression levels onto the same UMAP, indicating its enriched expression in tumor cells. (**C**) Spatial transcriptomic data from five HCC patients (HCC1–HCC5) confirm the predominant expression of PTK2 within annotated tumor regions across multiple cases. (**D**) Spatial transcriptomic profiling of PTK2 expression in one representative HCC sample (HCC-1). From left to right: three distinct tissue sections from patient HCC-1 are shown. For each section, top to bottom: H&E staining image, spatial cell type distribution, and spatial expression of PTK2 are presented, demonstrating its specific overexpression in tumor cell regions. (**E**) Box plot shows significantly elevated PTK2 mRNA levels in HCC tumors (*n* = 369) compared to normal liver tissues (*n* = 50) in the TCGA cohort. *** *p* < 0.001. (**F**) ROC curve evaluating the diagnostic value of PTK2 expression for distinguishing HCC from normal tissues.

Immunohistochemical (IHC) staining from the HPA showed that HCC tissues stained with CAB004036 exhibited moderate staining intensity, whereas adjacent normal liver tissues showed low staining (Figure 7A). Uniform Manifold Approximation and Projection (UMAP) analysis revealed that PTK2 was predominantly expressed in tumor tissues, with minimal expression in normal or peritumoral tissues (Figure 7B). Spatial transcriptomic data from five HCC patients in the HCCDB database consistently showed high PTK2 expression in tumor regions, while expression in non-tumor regions was significantly lower (Figure 7C). A representative single-cell transcriptomic profile from Patient HCC-1 is shown in Figure 7D. Other cases are provided in Figure S2.

Furthermore, analysis of RNA-seq data from the TCGA revealed that PTK2 mRNA levels were significantly higher in HCC samples (*n* = 369) than in normal liver tissues (*n* = 50) (Figure 7E). Figure S3 shows the differential gene expression profiles of HCC and normal liver tissues in the TCGA dataset.

These findings from multiple datasets consistently indicate upregulated expression of PTK2 in tumor tissues. Finally, the ROC curve showed an *AUC* of 0.809 for PTK2 (Figure 7F), suggesting its potential utility as a diagnostic biomarker for HCC. Although the ROC analysis yielded an *AUC* of 0.809, indicating fair diagnostic accuracy, this value suggests moderate discriminative power. Therefore, PTK2 alone may not be sufficient as a standalone biomarker for clinical diagnosis of HCC.

### 3.7. ZQ Suppresses Tumor Growth and Inhibits the PTK2/PI3K/AKT Signaling Pathway in a Murine HCC Model

**Figure 8:**
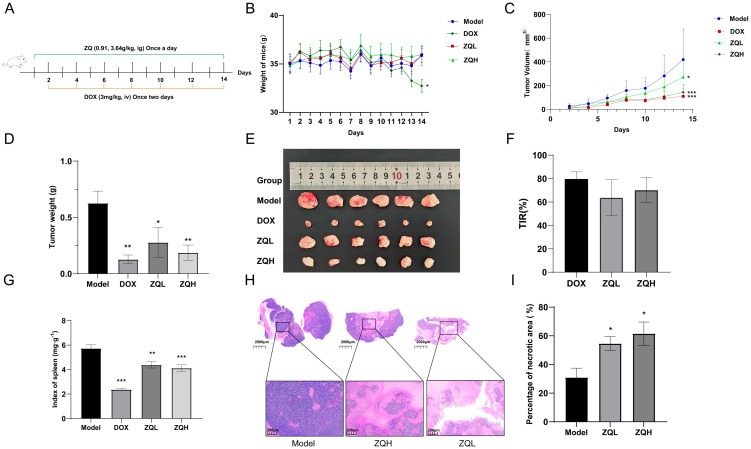
Effects of ZQ treatment on body weight, tumor burden, and spleen index in H22 tumor-bearing mice (*n* = 6). (**A**) Schematic of the treatment regimen. Mice were administered ZQ (0.91 or 3.64 g/kg, i.g., once daily) or DOX (3 mg/kg, i.v., every other day) for 14 consecutive days. (**B**) Body weights were recorded once daily during the entire course of treatment. (**C**) Tumor growth curves based on tumor volume measurements over time. ZQ treatment significantly inhibited tumor growth. (**D**) Final tumor weights measured at the end of the study. Both low and high doses of ZQ significantly reduced tumor weight compared to the Model group. (**E**) Representative images of excised tumor tissues from each group. (**F**) TIR% calculated for each treatment group. ZQ treatment achieved significant TIRs of 63.68% (ZQL) and 70.16% (ZQH). (**G**) Spleen index (mg/g), calculated as spleen weight normalized to body weight. (**H**) Representative H&E-stained sections of tumor tissues. Pale eosin-stained areas indicate necrotic regions. Scale bars: 2000 μm (overview), 200 μm (magnified view). (**I**) Quantification of tumor necrotic area percentage in each group. Both low and high doses of ZQ significantly increased tumor necrosis. Data are presented as mean ± SEM (*n* = 3 for panel (**I**)). Statistical significance was evaluated using one-way ANOVA followed by Tukey’s post hoc test. Statistical comparisons were made versus the model group: * *p* < 0.05, ** *p* < 0.01, *** *p* < 0.001.

To investigate the in vivo antitumor activity of ZQ, a subcutaneous H22 tumor-bearing mouse model was established. Mice received daily oral ZQ administration for 14 consecutive days (Figure 8A), and tumor volume was measured every other day. During the treatment period, animals in all groups maintained normal appetite. However, mice in DOX group exhibited reduced activity, general malaise, and significant weight loss (*p* = 0.0364, Figure 8B).

Tumor growth rates exhibited a descending trend across treatment groups: Control > ZQL > ZQH > DOX (Figure 8C). Compared with the Control group, ZQL and ZQH treatment significantly inhibited tumor growth in a dose-dependent manner (*p* = 0.0396 and *p* = 0.0086), as shown in Figure 8D,E. The TIR was 79.65% in the DOX group, 63.68% in the ZQL, and 70.16% in the ZQH (Figure 8F).

To assess immune effects, spleen indices were measured (Figure 8G). The DOX group showed a significant decrease (*p* < 0.001), indicating immunotoxicity. In contrast, the ZQL and ZQH showed milder reductions but remained significantly higher than DOX (*p* = 0.0024 and *p* < 0.001), suggesting that ZQ causes less immune suppression and has better safety.

Histological changes in tumor tissues were examined using H&E staining (Figure 8H). Tumors in the model group showed densely packed cells with deep nuclear staining, large nuclei, and predominantly round or spindle-shaped morphology, which are characteristic features of highly proliferative tumor cells. In contrast, tumors from the ZQL and ZQH displayed reduced cell density, lighter nuclear staining, and loosely arranged, irregularly shaped cells. Pronounced features of nuclear pyknosis and fragmentation were observed, along with areas of necrosis. Quantitative analysis showed that the necrotic area percentages in the ZQL and ZQH were significantly higher than those in the model group. Compared with the model group, the ZQL and ZQH showed significantly higher percentages of necrotic areas (*p* = 0.0497 and *p* = 0.0238; Figure 8I and Figure S4). These results suggest that ZQ effectively inhibits tumor cell proliferation and promotes tumor necrosis in a dose-dependent manner.

**Figure 9:**
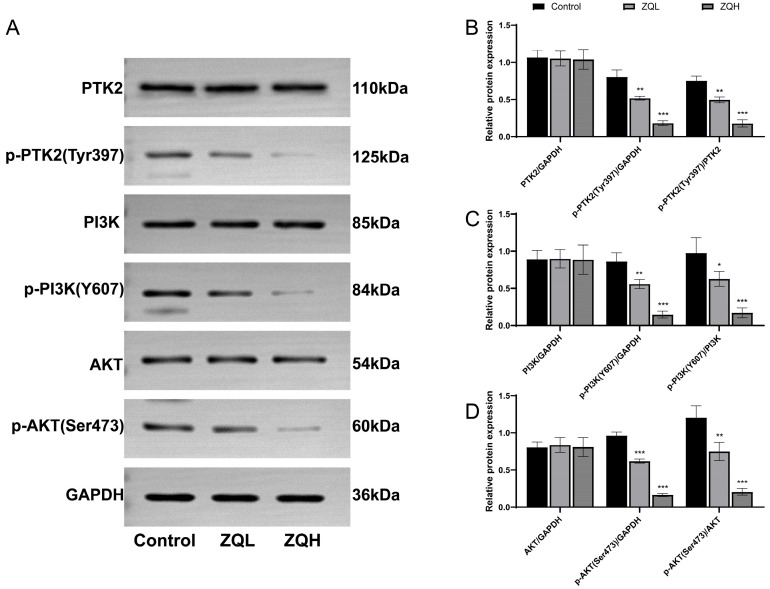
Effects of ZQ treatment on PTK2/PI3K/AKT signaling pathway in mouse tumor tissues (*n* = 3). (**A**) Representative Western blot images showing the expression of total and phosphorylated PTK2 (Tyr397), PI3K (Tyr607), and AKT (Ser473) proteins in control, ZQL, and ZQH. GAPDH served as the internal loading control. (**B**) Quantification of total and phosphorylated PTK2 (Tyr397), normalized to GAPDH, and the ratio of phosphorylated PTK2 (Tyr397) to total PTK2. (**C**) Quantification of total and phosphorylated PI3K (Tyr607), normalized to GAPDH, and the ratio of phosphorylated PI3K (Tyr607) to total PI3K. (**D**) Quantification of total and phosphorylated AKT (Ser473), normalized to GAPDH, and the ratio of phosphorylated AKT (Ser473) to total AKT. Data are expressed as mean ± SEM (*n* = 3). Statistical significance was evaluated using one-way ANOVA followed by Tukey’s post hoc test. Statistical significance compared to the control group: * *p* < 0.05, ** *p* < 0.01, *** *p* < 0.001.

Based on the identification of PTK2 as a core target and enrichment of the PI3K/AKT signaling pathway in network pharmacology analysis, we examined the regulatory effect of ZQ on this pathway in vivo. Representative cropped Western blot bands are shown in Figure 9A; full-length original blots are provided in Figure S5. Western blot analysis (Figure 9) revealed that the phosphorylation level of PTK2 at Tyr397 (p-PTK2) was significantly decreased in both ZQL and ZQH compared to the Control group (*p* = 0.0022 and *p* < 0.001). Meanwhile, total PTK2 protein levels remained unchanged. Similarly, phosphorylated PI3K (p-PI3K, Y607) and AKT (p-AKT, Ser473) levels were also markedly reduced in a dose-dependent manner (*p* = 0.0015 and *p* < 0.001 for p-PI3K; *p* = 0.0380 and *p* < 0.001 for p-AKT). No significant changes were observed in total PI3K or AKT expression. These results suggest that ZQ may exert anti-HCC effects by inhibiting the phosphorylation and activation of the PTK2/PI3K/AKT signaling axis.

### 3.8. ZQ Phytochemicals Exhibit Stable Binding to PTK2: Insights from Docking and MD Simulations

**Figure 10:**
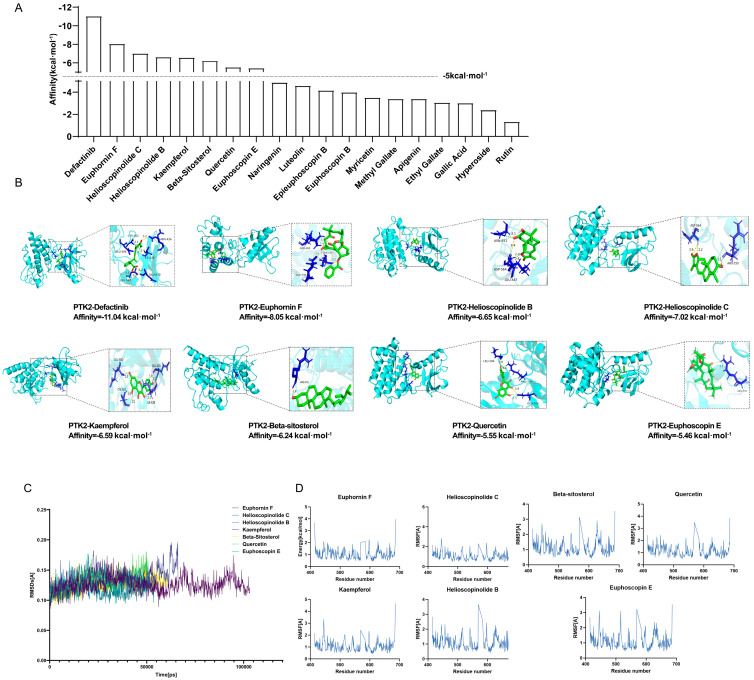
Molecular docking and MD simulations reveal stable binding of ZQ-derived active compounds to PTK2. (**A**) Binding affinities (kcal/mol) of 18 potential active compounds from ZQ with PTK2. The dashed line at −5 kcal/mol indicates the cutoff for strong binding potential. Seven compounds and Defactinib showed superior binding affinities stronger than −5 kcal/mol. (**B**) Representative binding poses of the top 7 compounds and Defactinib within the ATP-binding pocket of PTK2. Close-up views illustrate key interactions between the ligands and protein residues, confirming their docking at the active site. (**C**) RMSD trajectories of the PTK2–ligand complexes over 100 ns MD simulations. The low average RMSD values (<2.0 Å) indicate minimal conformational fluctuation and overall high stability of all seven complexes during the simulations. (**D**) RMSF profiles of PTK2 residues in complex with the 7 selected compounds. The low flexibility observed for most residues in the binding region further corroborates the stability of the protein-ligand interactions.

To evaluate the binding potential of ZQ-derived potential active compounds with PTK2, eighteen representative molecules and the PTK2 inhibitor Defactinib were subjected to molecular docking using AutoDock Vina. As shown in Figure 10A, several compounds exhibited strong binding affinities (below −5 kcal/mol), among which Euphornin F (−8.05 kcal/mol), Helioscopinolide C (−7.02 kcal/mol), Helioscopinolide B (−6.65 kcal/mol), Kaempferol (−6.59 kcal/mol), Beta-sitosterol (−6.24 kcal/mol), Quercetin (−5.55 kcal/mol), and Euphoscopin E (−5.46 kcal/mol) were ranked among the top binders. However, their binding affinities were less negative than that of Defactinib (−11.04 kcal/mol). These ligands were visualized in complex with PTK2 using PyMOL (Figure 10B), and all exhibited well-defined binding poses at the ATP-binding pocket.

To further assess the stability of these interactions, MD simulations were conducted for the seven ligands. The RMSD plots (Figure 10C) showed overall stability throughout the 100 ns simulation, with average RMSD values < 2.0 Å, indicating minimal conformational fluctuations of the protein–ligand complexes. Additionally, RMSF profiles (Figure 10D) revealed that most residues in the PTK2 binding region exhibited low flexibility, further supporting the stability of these interactions.

Collectively, these findings suggest that selected phytochemicals in ZQ can stably bind to PTK2, providing computational evidence for their potential to modulate PTK2-related signaling pathways.

## 4. Discussion

In this study, we employed a multi-disciplinary strategy to investigate the anti-HCC mechanisms of ZQ, integrating UHPLC-Q-Exactive Orbitrap HRMS-based phytochemical profiling, network pharmacology, MR analysis, in vivo experiments, and molecular modeling. This approach not only identified PTK2 as a key target but also highlighted the complex, multi-target action profile of this traditional herb.

ZQ, widely used in traditional Chinese medicine for treating inflammatory and neoplastic conditions, has demonstrated potential against HCC in previous studies [36]. However, these studies lacked systematic identification of its active constituents and the core therapeutic mechanisms. In our study, we identified 104 chemical constituents, including 31 flavonoids and 8 terpenoids, using the advanced technology platform UHPLC-Q-Exactive Plus Orbitrap HRMS. This method combines UHPLC’s separation power with high-resolution mass spectrometry, enabling precise mass measurements and rapid identification of compounds even without reference standards [37,38]. We identified several compounds, such as triptolide lactone A, norisoboldine, baicalin, chrysin, and silymarin, for the first time in ZQ, enriching its phytochemical profile and providing a foundation for exploring its pharmacological activities. The 70% ethanol extraction method was chosen for its balanced polarity, commonly used in previous studies for broad-spectrum extraction [39,40].

Network pharmacology revealed 18 bioactive compounds targeting 128 HCC-related proteins, with KEGG pathway enrichment indicating involvement in PI3K-AKT, EGFR resistance, and MAPK signaling. However, when we integrated topological analysis with MR analysis, PTK2 (also known as Focal Adhesion Kinase, FAK) emerged as a central node with robust causal support. PTK2 plays pivotal roles in integrin-mediated signaling, cell proliferation, adhesion, and migration [41,42], and is increasingly recognized as a driver of HCC progression and metastasis [43,44]. Bioinformatics analyses revealed elevated PTK2 expression in HCC tissues across multiple datasets. While its diagnostic value is moderate (*AUC* of 0.809), PTK2 should be considered as part of a broader diagnostic panel rather than as a standalone marker. Notably, MR analysis confirmed a stable, positive causal relationship between PTK2 expression and HCC risk, validating PTK2 as a potential therapeutic target. However, due to the molecular heterogeneity of HCC, PTK2′s diagnostic and prognostic value may be limited in isolation but could be enhanced in combination with other biomarkers for improved risk stratification and predictive accuracy.

The ZQ dosage used in this study for mice was calculated based on clinical human doses. According to the Chinese Materia Medica [45], ZQ has low toxicity, with a clinical daily dose of 3–9 g. Assuming a standard human weight of 60 kg, this corresponds to approximately 50–150 mg/kg. Using a human-to-mouse body surface area conversion factor of 9.1, the equivalent mouse dose is 0.455–1.365 g/kg. On this basis, 0.91 g/kg was selected as the low dose and 3.64 g/kg as the high dose in the experiment. The low dose (0.91 g/kg) corresponds to 232 mg/kg of the ZQ extract, while the high dose (3.64 g/kg) is four times the low dose and corresponds to 928.2 mg/kg of the extract. In addition, we referenced a study by W. Duan et al. (2025) [10], which reported ZQ doses ranging from 0.91 g/kg to 3.64 g/kg for antitumor effects in lung cancer models.

To assess the safety of ZQ extract, we conducted a preliminary evaluation based on body weight and spleen index. Although a transient weight decrease was observed in the high-dose group, likely due to tumor burden or a temporary drug response. However, no mortality or severe distress was observed. Several toxicological studies have confirmed the safety of ZQ. Deshpande et al. reported no toxicity or organ changes in mice administered a 5 g/kg dose of ZQ aqueous extract [46]. Similarly, Duan et al. concluded that long-term ZQ treatment did not cause liver damage, despite fluctuations in liver index [10]. Furthermore, Saleem et al. reported an LD_50_ for ZQ methanol extract greater than 2000 mg/kg, with no organ damage [47]. Taken together, these findings support the notion that ZQ extract has a wide safety margin, making the weight changes observed in our study unlikely to reflect organ toxicity.

The dosing regimen for DOX was also based on clinical data and adjusted for mice. The DOX dosage was determined according to our previous study [48], which ensured effective inhibition of tumor growth in the H22 tumor-bearing mouse model while maintaining toxicity within an acceptable range. In this study, DOX served as a qualitative positive control. Its primary role was to validate the experimental system and to provide a benchmark for evaluating the antitumor activity of ZQ.

In vivo experiments further supported the involvement of PTK2 in the anti-HCC effects of ZQ. Oral administration of ZQ suppressed tumor growth in H22-bearing mice and was associated with less immune suppression than DOX. Western blot analysis showed that ZQ significantly reduced PTK2 phosphorylation at Tyr397, accompanied by decreased phosphorylation of PI3K and AKT, without altering total protein levels. Given the central role of the PI3K/AKT pathway in apoptosis, metabolic reprogramming, and epithelial–mesenchymal transition [49,50], these results suggest that ZQ-mediated modulation of this pathway may contribute to antitumor activity beyond growth inhibition alone. Although apoptosis-related proteins were not assessed, the observed signaling profile is consistent with a growth-suppressive, rather than acutely cytotoxic, mechanism. Further evaluation of apoptosis markers, cell cycle regulators, and downstream effectors such as mTOR will be required to clarify the underlying cellular processes.

Our data further indicate that the antitumor activity of ZQ differs mechanistically from that of classical cytotoxic agents such as DOX. Histopathological analysis revealed increased intratumoral necrosis following ZQ treatment, without rapid or pronounced tumor shrinkage, a pattern more consistent with delayed tumor progression than direct cytotoxicity. Consistent with this observation, ZQ selectively inhibited PTK2/PI3K/AKT phosphorylation while preserving total protein expression. As PTK2 activation promotes proliferation and apoptosis resistance, its inhibition may limit tumor expansion and reduce adaptive stress tolerance, with necrosis likely occurring as a secondary consequence of impaired signaling rather than direct cell killing. These features suggest that ZQ may be better suited for adjuvant or long-term maintenance strategies aimed at restraining tumor progression.

This study investigates the anti-HCC mechanism of ZQ from a systems perspective. The results indicate that PTK2 is a central node within the regulatory network, but the anti-tumor effects of ZQ are likely due to coordinated actions among multiple bioactive components and their regulation of related targets. Network analysis suggests that PTK2, along with EGFR, MAPK14, and ESR2, constitutes key regulatory nodes. EGFR, as a receptor tyrosine kinase, regulates cell survival and proliferation signaling, and its inhibition alongside PTK2 may enhance suppression of downstream PI3K/AKT and MAPK pathways, thereby reducing drug resistance [51]. MAPK14 (p38 MAPK) regulates stress responses and inflammation, suggesting that ZQ may impact the tumor microenvironment and induce stress-related apoptosis [52]. ESR2 is involved in hormone signaling and may be relevant to certain HCC subtypes [53]. Molecular docking and MD simulations further showed that multiple ZQ-derived compounds can stably bind to PTK2, supporting potential synergistic interactions at a common target. These findings provide evidence for a multi-component, multi-target, multi-pathway regulatory model, although the functional significance of these interactions requires further validation.

Molecular docking and MD simulations revealed that seven active ZQ components, including Euphornin F and Quercetin, bind stably to PTK2′s ATP-binding pocket. In vivo, ZQ downregulated p-PTK2 (Tyr397) levels in tumor tissues in a dose-dependent manner, validating PTK2 modulation. However, due to the conserved nature of kinase catalytic domains, off-target effects on other kinases cannot be excluded. Future studies should incorporate Surface Plasmon Resonance (SPR) or Cellular Thermal Shift Assay (CETSA) to quantify binding kinetics and confirm specificity.

This study employs MR analysis to address the limitations of traditional network pharmacology in causal inference, providing causal evidence for ZQ’s anti-HCC effects. Traditional network pharmacology identifies potential drug targets but cannot confirm their direct causal impact on diseases [54]. By using genetic variants as instruments, we minimized environmental confounders and provided robust causal evidence linking PTK2 to HCC risk. This approach demonstrates how ethnopharmacological knowledge can be integrated with systems biology, bridging traditional herbal knowledge and modern pharmacology. Such integrative methods offer promising pathways for developing scientifically validated, mechanism-based phytomedicines.

However, several limitations should be acknowledged. First, the H22 subcutaneous mouse model does not fully represent human HCC’s complex etiology, such as chronic inflammation, hepatic fibrosis, and immune heterogeneity. Future studies should use orthotopic transplantation or patient-derived xenograft models to better replicate the human tumor microenvironment [55]. Second, MR analysis was limited by the small HCC GWAS dataset, leading to potential power and bias issues. Moreover, the analysis used blood-derived cis-eQTLs, which may not fully reflect liver- or tumor-specific regulation. Further studies with larger, multi-ethnic GWAS datasets and liver-specific eQTLs will be essential for refining causal inference.

Although we suggest PTK2 as the core therapeutic target of ZQ, this conclusion is mainly based on bioinformatics and pathway-level analysis, lacking direct functional validation. Future studies should include PTK2 inhibitors as controls to assess synergy with ZQ. Gene knockdown or overexpression experiments should confirm whether ZQ’s effects depend on PTK2 expression, providing a clearer understanding of the PTK2/PI3K/AKT pathway’s role in ZQ’s anti-tumor activity and supporting preclinical translation.

In summary, this study identifies PTK2 as a core therapeutic target of ZQ in HCC and shows that ZQ inhibits the PTK2/PI3K/AKT pathway, exerting multi-level anti-tumor effects. The polycomponent, multi-pathway profile of ZQ suggests its potential as a systems-level therapeutic, supporting its development as a promising anti-HCC agent derived from traditional medicine.

## 5. Conclusions

In summary, this study systematically elucidated the anti-HCC mechanism of ZQ using an integrative strategy combining UHPLC-Q-Exactive Orbitrap HRMS, network pharmacology, MR analysis, and experimental validation. PTK2 was identified as a core therapeutic target. While PTK2 is central to ZQ’s therapeutic action, the complexity of its pharmacological profile suggests that other key targets, including EGFR, MAPK14, and ESR2, may also contribute significantly to ZQ’s anti-HCC effects, highlighting its multi-target nature. In vivo experiments confirmed that ZQ significantly suppressed tumor growth by modulating the PTK2/PI3K/AKT signaling pathway. These findings provide mechanistic insights into ZQ’s multi-target pharmacological actions and support its potential as a promising ethnopharmacological candidate for HCC therapy. However, further experimental validation is needed to fully understand the contributions of other identified targets.

## Data Availability

The raw data supporting the conclusions of this article will be made available by the authors on request.

## Supplementary Materials

The following supporting information can be downloaded at: https://www.mdpi.com/article/10.3390/cimb48020213/s1.
