# Supplementary material for: Elucidating the PTK2-Targeted Anti-Hepatocellular Carcinoma Effects of Euphorbia helioscopia L. via Integrated Network Pharmacology, Mendelian Randomization, and Experimental Validation
Source: Curr Issues Mol Biol. 2026 Feb 14;48(2):213. doi: 10.3390/cimb48020213 (PMC12939734; doi:10.3390/cimb48020213)
Supplement: Supplementary file 1 [file cimb-48-00213-s001.zip › cimb-4122984-supplementary.pdf]

Supplementary Information

Table S1. Identification of chemical constituents in ZQ

No.	t_R/min	molecular formula	Molecular ion peak	Actual m / z	theory m / z	MS² fragment ions	Error / × 10⁻⁶	Identification results	Compound category
1	0.32	$C_{13}H_{25}NO_3$	$[M+H]^+$	171.1161	171.1168	171.1159, 141.0685	4.27	1,6,7-Trimethylnaphthalene	others
2	0.59	$C_{15}H_{10}O_6$	$[M-H]^-$	286.0472	286.0471	65.0017, 107.0123, 121.0277, 133.0279	-0.17	Echinacoside	flavonoids
3	0.70	$C_{15}H_{10}O_6$	$[M+H]^+$	287.0533	287.0550	241.0480, 68.9975, 105.0333	5.82	Kaempferol	flavonoids
4	0.90	$C_6H_{14}O_6$	$[M-H]^-$	182.0785	182.0775	59.0124, 113.0230, 73.0279, 89.0228, 101.0228, 149.0442, 163.0606	-5.44	Sorbitol	glycosides
5	0.91	$C_{12}H_{22}O_{11}$	$[M-H]^-$	341.1157	341.1148	59.0123, 71.0123, 89.0228, 101.0228	-2.49	Sucrose	glycosides
6	0.92	$C_6H_{14}N_4O_2$	$[M+H]^+$	174.1111	174.1108	158.0916, 116.0702, 130.0969, 175.1180	-1.90	DL-Arginine	amino acid
7	0.92	$C_6H_{12}O_7$	$[M-H]^-$	195.0497	195.0499	59.0123, 71.0123, 75.0072, 89.0228, 177.0390	0.82	Gluconic acid	organic acids
8	0.92	$C_{12}H_{22}O_{11}$	$[M-H]^-$	341.1077	341.1078	59.0123, 71.0123, 89.0228	0.38	D-(+)-Trehalose	glycosides
9	0.99	$C_9H_{13}NO_6$	$[M+H]^+$	232.0737	232.0733	126.0534, 168.0645, 186.0753	-1.77	N2-Succinyl-L-glutamate-5-semialdehyde	amino acid

No.	t_R/min	molecular formula	Molecular ion peak	Actual m / z	theory m / z	MS² fragment ions	Error / × 10⁻⁶	Identification results	Compound category
10	0.99	$C_7H_{13}NO_3$	$[M-H]^-$	159.0900	159.0900	74.0231, 114.0909	-0.25	Isovaleroylglycine	others
11	1.06	C20H28O5	$[M-H]^-$	347.1836	347.1853	329.1766, 317.1752, 299.1648	5.01	Ingenol	terpenes
12	1.09	$C_{11}H_{21}NO_{10}S_3$	$[M-H]^-$	422.0239	422.0243	195.9724, 422.0240	0.92	Glabellin	
13	1.14	$C_6H_{12}O_6$	$[M-H]^-$	179.0547	179.0550	59.0123, 71.0123, 75.0072, 179.0548	1.22	D-Ajecose	glycosides
14	1.26	$C_6H_8O_7$	$[M-H]^-$	192.0265	192.0255	85.0281, 102.9474, 111.0075	-4.95	Citric acid	organicacids
15	1.27	$C_9H_{11}NO_3$	$[M+H]^+$	181.0733	181.0730	136.0750, 91.0542, 123.0435, 119.0486	-1.88	L-Tyrosine	amino acid
16	1.34	$C_{10}H_{13}N_5O_5$	$[M-H]^-$	282.0811	282.0833	133.0140, 150.0406	7.69	Guanosine	others
17	1.51	$C_8H_9NO_4$	$[M-H]^-$	183.0526	183.0517	120.0437, 138.0545, 182.0443	-4.97	3,5-Dihydroxyphenylglycine	amino acid
18	1.59	$C_{11}H_{11}NO_3$	$[M+H]^+$	206.0733	206.0729	206.0798, 160.0746	-2.09	5-Methoxyindoleacetic acid	others
19	1.65	$C_7H_6O_5$	$[M-H]^-$	169.0127	169.0131	125.0228, 126.0261, 124.0152	2.37	Gallic acid	organic acids
20	1.67	$C_{11}H_{15}N_5O_4$	$[M+H]^+$	282.1119	282.1113	136.0611, 119.0351, 282.1177	-2.16	2'-O-Methyladenosine	others
21	1.71	$C_7H_{12}O_6$	$[M-H]^-$	191.0550	191.0545	173.0075, 93.0328, 111.0071, 129.0383	2.41	Quinic acid	organic acids
22	2.02	$C_{10}H_{13}NO_2$	$[M+H]^+$	180.1017	180.1019	134.0964, 117.0701, 91.0546, 79.0547	0.12	Salsolinol	Alkaloids
23	2.21	$C_{10}H_{15}NO$	$[M+H]^+$	165.1148	165.1143	79.0545, 103.0541	-3.15	Hordenine	Alkaloids
24	2.23	$C_9H_{11}NO_2$	$[M-H]^-$	164.0784	164.0783	61.9868, 72.0091	-1.04	L-Phenylalanine	amino acid
25	2.59	$C_9H_{17}NO_5$	$[M+H]^+$	219.1101	219.1097	98.0235, 124.0751, 184.0958, 202.1061	-2.15	Pantothenic acid	organic acids
26	3.12	$C_7H_6O_4$	$[M-H]^-$	153.0177	153.0182	109.0278, 153.0177	3.46	Gentisic acid	organic acids
27	3.63	$C_{27}H_{22}O_{18}$	$[M-H]^-$	633.0747	633.0722	300.9979, 275.0189	-3.85	Euphorbia tannin B	phenols

No.	t_R/min	molecular formula	Molecular ion peak	Actual m / z	theory m / z	MS^2 fragment ions	Error / × 10^{-6}	Identification results	Compound category
28	3.63	$C_{16}H_{18}O_9$	$[M-H]^-$	353.086	353.0867	135.0435, 179.0334, 191.0546	1.72	Chlorogenic acid	organic acids
29	3.65	$C_{16}H_{18}O_9$	$[M-H]^-$	353.0863	353.0867	135.0435, 191.0546	1.07	Neochlorogenic acid	organic acids
30	3.81	$C_{16}H_{18}O_9$	$[M-H]^-$	353.0873	353.0878	93.0332, 135.0441, 179.0343, 191.0554	0.13	Poncirin	flavonoids
31	4.11	$C_{10}H_7NO_3$	$[M+H]^+$	189.0420	189.0416	65.0390, 190.0483	-2.33	Kynurenic acid	organic acids
32	4.23	$C_8H_8O_5$	$[M-H]^-$	183.0284	183.0288	95.0122, 168.0049, 123.0071	1.97	Methyl gallate	organic acids
33	4.31	$C_{27}H_{22}O_{18}$	$[M-2H]^{2-}$	632.0801	632.0787	125.0224, 169.0127, 300.9979, 633.0716	-2.18	Corilagin	phenols
34	4.35	$C_{11}H_{10}O_5$	$[M+H]^+$	223.0599	223.0601	107.0490, 162.0304, 190.0250, 208.0356	0.85	Isofraxidin	coumarins
35	4.48	$C_9H_{11}NO_2$	$[M+H]^+$	165.0784	165.0783	120.0803, 131.0485	-1.03	DL-Phenylalanine	amino acid
36	4.57	$C_{21}H_{20}O_{13}$	$[M-H]^-$	479.0803	479.0820	316.0212, 271.0239, 259.0241	3.52	Myricetin-3-galactoside	flavonoids
37	4.59	$C_{15}H_{10}O_8$	$[M+H]^+$	319.0448	319.0448	273.0373, 245.0429, 217.0485, 153.0173	0.13	Myricetin	flavonoids
38	4.61	$C_{21}H_{20}O_{13}$	$[M+H]^+$	481.0898	481.0887	85.0285, 319.0427	-2.33	Myricetin-3-O-β-D-galactopyranoside	flavonoids
39	4.66	$C_{21}H_{20}O_{12}$	$[M-H]^-$	479.0846	479.0820	316.0212, 151.0030	-5.41	Myricetin-3'-O-β-D-glucoside	flavonoids
40	4.71	$C_{27}H_{30}O_{16}$	$[M-H]^-$	609.1448	609.1444	285.0393, 227.0335, 255.0290, 301.0331	-0.71	Rutin	flavonoids
41	4.71	$C_{27}H_{29}O_{16}$	$[M-H]^-$	609.1444	609.1435	301.0331, 151.0026	1.40	Quercetin-3-O-rutinoside	flavonoids
42	4.73	$C_{10}H_8O_5$	$[M+H]^+$	209.0444	209.0430	194.0199, 163.0380, 149.0224	6.52	Circularin	others
43	4.80	$C_{21}H_{20}O_{12}$	$[M+H]^+$	465.1045	465.1028	257.0427, 153.0173	-3.76	Hyperin	flavonoids

No.	t_R/min	molecular formula	Molecular ion peak	Actual m / z	theory m / z	MS^2 fragment ions	Error / × 10^{-6}	Identification results	Compound category
44	4.81	$C_{21}H_{20}O_{12}$	$[M-H]^-$	463.0867	463.0871	300.0264, 271.0240, 255.2910, 151.0019	0.73	Isoquercitrin	flavonoids
45	4.83	$C_{14}H_6O_8$	$[M-H]^-$	301.0057	301.0051	300.9978, 257.0084, 201.0180	-1.93	Ellagic acid	organic acids
46	4.83	$C_{15}H_{10}O_7$	$[M-H]^-$	301.0344	301.0342	273.0400, 107.0123, 193.0124, 151.0022	0.66	Quercetin	flavonoids
47	4.89	$C_{28}H_{24}O_{16}$	$[M+H]^+$	617.1108	617.1137	153.0177, 303.0495, 97.0288	4.72	2-O-Galloylhyperin	flavonoids
48	4.90	$C_{21}H_{18}O_{13}$	$[M-H]^-$	477.0642	477.0664	107.0119, 125.0229, 151.0015, 301.0325	4.55	Quercetin-3-O-glucuronide	flavonoids
49	4.90	$C_{21}H_{20}O_{13}$	$[M-H]^-$	479.0816	479.0820	479.0816, 151.0021, 317.0291	-0.79	Gossypin	glycosides
50	4.91	$C_{13}H_{18}O_2$	$[M+H]^+$	206.1301	206.1298	55.0547, 79.0546, 65.0389, 107.0853	-1.79	Etrogol	others
51	4.92	$C_9H_{10}O_5$	$[M-H]^-$	198.0523	198.0514	125.0228, 111.0071, 197.0441	-4.39	Syringic acid	organic acids
52	4.94	$C_9H_{10}O_5$	$[M-H]^-$	197.044	197.0444	111.0071, 125.0228, 169.0127	1.82	Ethyl gallate	organic acids
53	5.01	$C_{20}H_{18}O_{11}$	$[M+H]^+$	435.0844	435.0833	153.0172, 195.0286	-2.51	Quercetin-3-arabinoside	flavonoids
54	5.03	$C_{21}H_{20}O_{11}$	$[M-H]^-$	447.1000	447.0990	107.0115, 225.0289, 284.0314, 447.0917	-2.17	Trifolirhizin	flavonoids
55	5.04	$C_{15}H_8O_5$	$[M-H]^-$	267.0276	267.0288	239.0336, 267.0288	4.38	Coumarin	coumarins
56	5.05	$C_{21}H_{20}O_{11}$	$[M-H]^-$	447.0921	447.0917	227.0336, 285.0387, 255.0289	1.05	Astragalin	flavonoids
57	5.14	$C_{13}H_{14}N_2O_3$	$[M-H]^-$	245.0999	245.0995	203.0813, 159.0906, 142.0648, 116.0489	-1.75	N-Acetyl-DL-tryptophan	amino acid
58	5.14	$C_{25}H_{24}O_{12}$	$[M-H]^-$	515.1262	515.1253	135.0436, 161.0230, 173.0440, 191.0549	-1.79	4,5-Dicaffeoylquinic acid	organic acids

No.	t_R/min	molecular formula	Molecular ion peak	Actual m / z	theory m / z	MS² fragment ions	Error / × 10⁻⁶	Identification results	Compound category
59	5.14	$C_{21}H_{22}O_{11}$	$[M-H]^-$	449.1078	449.1056	287.0551, 449.1057, 151.0022, 135.0435	4.79	Eriodictyol-7-O-glucoside	flavonoids
60	5.16	$C_{15}H_{12}O_5$	$[M+H]^+$	273.0679	273.0674	91.0542, 273.1836	-1.87	Naringenin	flavonoids
61	5.16	$C_{13}H_{14}N_2O_3$	$[M+H]^+$	247.1075	247.1077	118.0647, 130.0645, 142.0641	0.72	N-Acetyltryptophan	amino acid
62	5.41	$C_{21}H_{22}O_{10}$	$[M-H]^-$	433.1135	433.1129	271.0603, 151.0021	-1.22	Naringenin-7-O-β-D-glucoside	flavonoids
63	5.43	$C_{21}H_{18}O_{11}$	$[M-H]^-$	445.0844	445.0833	251.0335, 269.0448	-2.31	Baicalin	flavonoids
64	5.73	$C_{15}H_{12}O_6$	$[M-H]^-$	287.0628	287.0625	135.0436, 151.0022, 287.0552	-1.05	Eriodictyol	flavonoids
65	5.99	$C_{11}H_{21}NOS$	$[M+H]^+$	215.1338	215.1336	55.0547, 72.0810, 84.0809, 86.0967	-1.16	Cyclolinopeptide	others
66	6.05	$C_{15}H_{10}O_5$	$[M-H]^-$	269.0523	269.0519	151.0021, 181.0640, 225.0548, 117.0330, 107.0123, 269.0446	-1.41	Wood flavonoids	flavonoids
67	6.12	$C_{20}H_{28}O$	$[M-H]^-$	347.1852	347.1866	285.1852, 303.1955, 329.1744	4.00	Euphelionolide A	terpenes
68	6.17	$C_{15}H_{10}O_6$	$[M+H]^+$	285.0392	285.0394	133.0275, 107.0121, 151.0022	0.51	Luteolin	flavonoids
69	6.29	$C_{15}H_{10}O_5$	$[M-H]^-$	269.0445	269.0444	181.0640, 159.0438, 151.0021, 117.0329, 107.0123, 225.0550	-3.59	Apigenin	flavonoids
70	6.35	$C_{18}H_{32}O_5$	$[M-H]^-$	327.2244	327.2238	171.1014, 185.1163, 211.1329, 327.2166	-1.86	Corchorifatty acid F	organic acids
71	6.37	$C_{16}H_{26}O$	$[M+H]^+$	234.1978	234.1974	147.1156, 133.1003, 107.0851, 83.0854	-1.96	2,6-Di-tert-butyl-4-ethylphenol	phenols
72	6.47	$C_{17}H_{12}O_8$	$[M+H]^+$	344.0527	344.0524	109.02771, 79.0180, 55.0183	-0.67	5,3',4'-Trihydroxy-3-methoxy-6,7-methylenedioxyflavone	flavonoids
73	6.49	$C_{15}H_{10}O_4$	$[M-H]^-$	253.0497	253.0495	209.1535, 143.0486, 181.0647	0.55	Chrysin	flavonoids
74	6.73	$C_{11}H_{16}O_2$	$[M+H]^+$	181.1229	181.1223	107.0853, 121.1007, 135.1161, 145.1004	-3.31	Olivetol	others
75	6.79	$C_{20}H_{26}O_4$	$[M+H]^+$	331.1877	331.1904	81.0703, 287.1251	8.24	Dicyclohexyl phthalate	others
76	6.83	$C_{20}H_{26}O$	$[M+H]^+$	331.1897	331.1899	285.1826, 185.1309	0.75	Helioscopinolide C	terpenes

No.	t_R/min	molecular formula	Molecular ion peak	Actual m / z	theory m / z	MS² fragment ions	Error / × 10⁻⁶	Identification results	Compound category
77	6.89	$C_{20}H_{26}O_4$	$[M-H]^-$	329.1768	329.1747	311.1652, 205.1221	-6.29	Euphcopenoid A	terpenes
78	6.95	$C_{16}H_{12}O_5$	$[M+H]^+$	285.0679	285.0672	270.0507, 285.0740	-2.53	Glycitein	flavonoids
79	7.08	$C_{17}H_{14}O_6$	$[M-H]^-$	313.0706	313.0706	313.0707, 107.0123	0.03	Cirsiliol	flavonoids
80	7.45	$C_{30}H_{48}O_4$	$[M+H]^+$	473.3565	473.3625	69.0701, 271.2040	12.59	Nigragillin	coumarins
81	7.64	$C_{20}H_{26}O_3$	[M+H]+	315.1877	315.1866	227.1418, 279.1724, 297.1832, 315.1934	-3.24	Cafestol	others
82	7.68	$C_{18}H_{39}NO_3$	$[M+H]^+$	318.2983	318.3002	60.0449, 282.2775, 300.2881	6.06	Phytosphingosine	others
83	7.72	$C_{27}H_{40}O_8$	$[M+H]^+$	493.2718	493.2715	493.2796, 93.0696	-0.63	Echinalin P	others
84	7.80	$C_9H_8O_3$	$[M-H]^-$	163.0401	163.0392	117.0335, 119.0502, 162.0316	-0.53	p-Hydroxycinnamic acid	organic acids
85	8.13	$C_{30}H_{42}O_7$	$[M+H]^+$	515.2999	515.3003	299.1992	0.78	Maltolactone B	others
86	8.26	$C_{16}H_{30}O_4$	$[M+H]^+$	286.2139	286.2136	67.0546, 95.0491	-0.98	Hexadecanedioic acid	organic acids
87	8.44	$C_{25}H_{42}N_4O_{14}$	$[M+H]^+$	623.2692	623.2721	503.2369, 623.2787	4.62	Aloesin	others
88	9.49	$C_{20}H_{26}O$	$[M+H]^+$	283.2034	283.2040	265.1932, 241.1573, 189.1261, 121.1007	2.33	19-Norpregna-1,3,5(10),20-tetraen-3-ol	steroids
89	9.49	$C_{23}H_{36}O_2$	$[M-H]^-$	343.2644	343.2631	209.1317, 243.1735	-3.79	(3S,4aR,9aS,9bS)-4a-Hydroxy-2-methyl-3-[(1E)-1-nonenyl]-4a,9,9a,9b-tetrahydrocyclopenta[f]chromen-5(3H)-one	terpenes
90	9.53	$C_{33}H_{44}O_9$	$[M+Na]^+$	607.2872	607.2872	105.0332, 283.2032, 365.2070, 487.2421	-0.05	Euphornin	terpenes
91	9.54	$C_{12}H_{12}$	$[M+H]^+$	156.0934	156.0930	129.0694, 157.1017, 155.0842	-2.24	2,6-Dimethylnaphthalene	others

No.	t_R/min	molecular formula	Molecular ion peak	Actual m / z	theory m / z	MS² fragment ions	Error / × 10⁻⁶	Identification results	Compound category
92	9.86	$C_{20}H_{35}NO_2$	$[M+H]^+$	322.2728	322.2740	322.2729, 304.2605, 67.0545, 121.1006	3.69	α-Linolenoyl ethanolamide	others
93	10.34	$C_{27}H_{30}O_3$	$[M+H]^+$	403.2261	403.2249	281.8829, 253.0112	-3.10	Gombapyrone E	others
94	11.93	$C_{15}H_{12}O_5$	$[M+H]^+$	273.0753	273.0757	107.0492, 154.0216, 172.0328, 255.0620	0.15	Naringin	flavonoids
95	12.10	$C_{20}H_{38}O_4$	$[M+H]^+$	342.2765	342.2757	71.0859, 95.0854, 109.1009	-2.28	Eicosanedioic acid	organic acids
96	12.72	$C_{18}H_{30}O_2$	$[M+H]^+$	279.2312	279.2319	95.0855, 67.0546, 223.1679, 109.1099	2.47	α-Linolenic acid	organic acids
97	16.06	$C_{13}H_{25}NO_3$	$[M+H]^+$	244.1904	244.1907	84.0444, 55.0547	1.52	n-Decanoylglycine	organic acids
98	16.38	$C_{19}H_{32}O_2$	$[M+H]^+$	292.2397	292.2389	55.0547, 67.0546, 93.0699, 107.0852	-2.74	Methyl linolenate	others
99	17.13	$C_{15}H_{10}O_5$	$[M+H]^+$	271.0595	271.0601	145.0283, 121.0288, 67.0184, 153.0180	0.23	Genistein	flavonoids
100	17.82	$C_{30}H_{46}O_3$	$[M+H]^+$	455.3523	455.3520	437.4000, 191.1000	-0.63	Triptolide	terpenes
101	19.79	$C_{22}H_{43}NO$	$[M+H]^+$	337.3339	337.3326	321.3127, 303.3043	-3.91	Erucamide	others
102	21.15	$C_{17}H_{24}O_3$	$[M+H]^+$	277.1795	277.1798	137.0596, 213.1635, 231.1737, 249.1844	0.12	Gingerol	phenols
103	24.58	$C_6H_{14}O_5$	$[M-H]^-$	166.0846	166.0846	57.0332, 85.0278, 87.0072, 89.0229	0.12	D-Fucitol	glycosides
104	28.32	$C_{30}H_{48}O_3$	$[M-H]^-$	439.3571	439.3569	107.0857, 119.0856, 103.1793	-0.04	Oleanolic acid	terpenes

Table S2. 18 potential active compounds of ZQ.

No,	Mol ID	Molecule Name	OB (%)	DL
1	MOL000422	Kaempferol	41.88	0.24
2	MOL004328	Naringenin	59.29	0.21
3	MOL000006	Luteolin	36.16	0.25
4	MOL000098	Quercetin	46.43	0.28
5	MOL000358	Beta-sitosterol	36.91	0.75
6	MOL000415	Rutin	3.20	0.68
7	MOL002008	Myricetin	13.75	0.31
8	MOL004368	Hyperoside	6.94	0.77
9	MOL001330	Gallic acid	28.63	0.28
10	MOL001906	Methyl gallate	30.91	0.05
11	MOL001331	Ethyl gallate	48.00	0.29
12	MOL000006	Apigenin	41.89	0.28
13	MOL013389	Epieuphoscopin B	30.07	0.76
14	MOL013401	Euphornin F	83.86	0.78
15	MOL013404	Euphoscopin B	30.07	0.75
16	MOL013407	Euphoscopin E	79.84	0.77
17	MOL013415	Helioscopinolide B	50.03	0.44
18	MOL013416	Helioscopinolide C	42.53	0.48

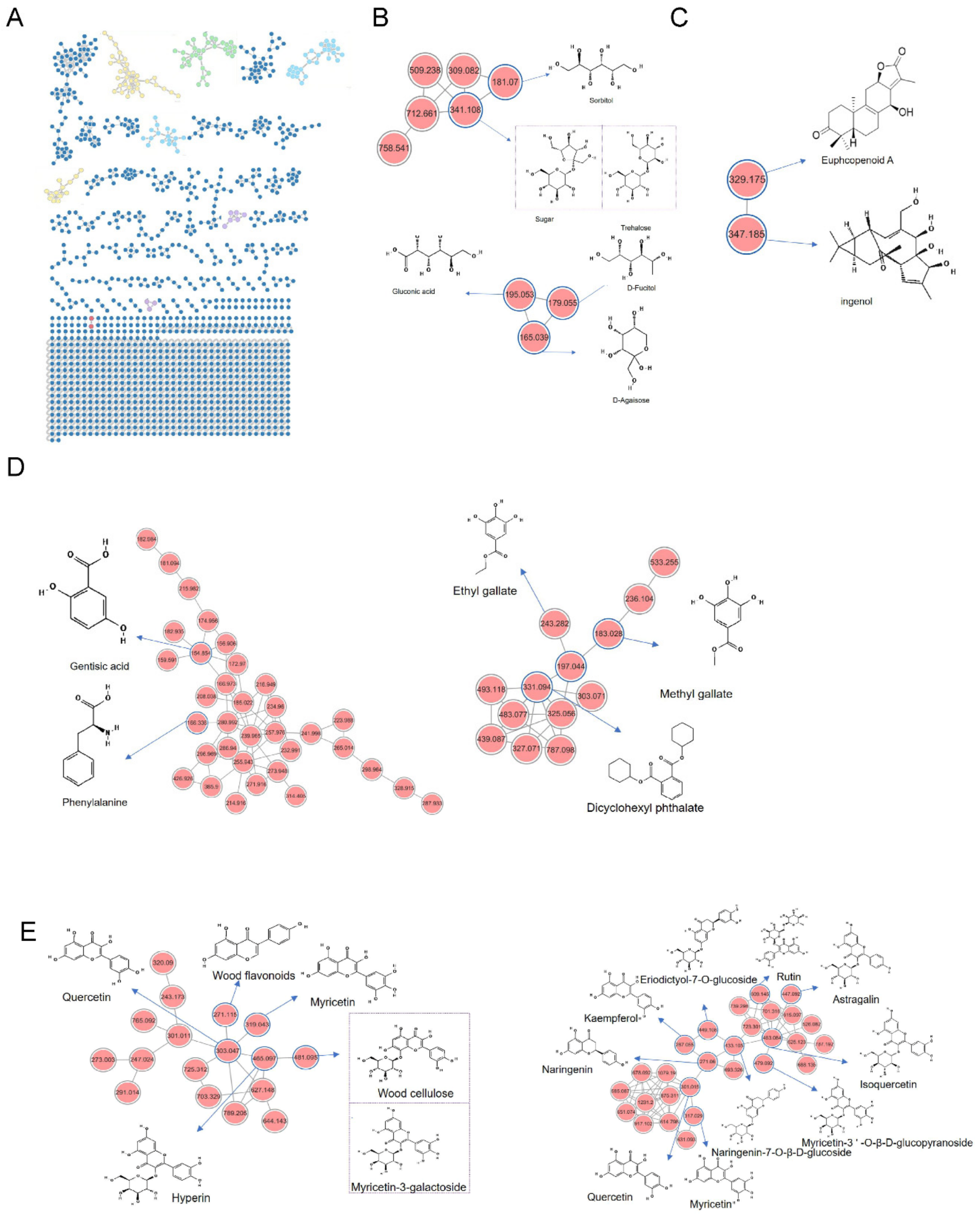

Figure S1. Molecular networking analysis and compound identification of ZQ ethanol extract.

(A) Molecular network analysis of compound clusters classified from ZQ.

(B) Enlarged molecular network of glycoside-type compounds (highlighted as the light purple cluster in the network).

(C) Enlarged molecular network of terpene-type compounds (highlighted as the light red cluster in the network).

(D) Enlarged molecular network of organic acid-type compounds (highlighted as the light yellow cluster in the network).

(E) Enlarged molecular network of flavonoid-type compounds (highlighted as the light purple cluster in the network).

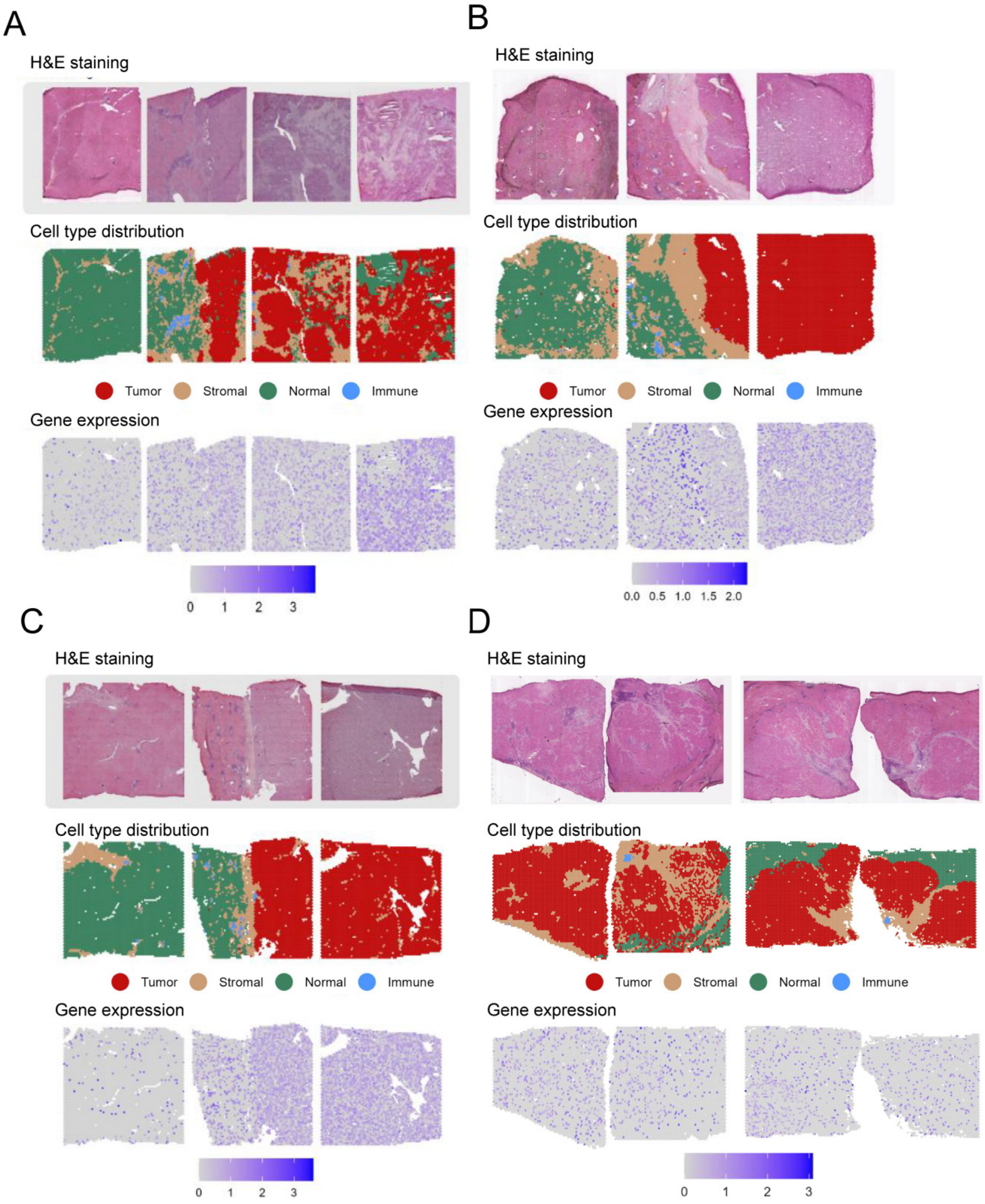

Figure S2. Spatial transcriptomic profiling of PTK2 gene expression across four hepatocellular carcinoma (HCC) patient samples: HCC-2 (A), HCC-3 (B), HCC-4 (C), and HCC-5 (D).

Each panel includes H&E staining images (top), spatial cell type distribution (middle), and spatial expression of the PTK2 gene (bottom). Cell types are annotated as tumor (red), stromal (brown), normal (green), and immune (blue). Gene expression is represented by a blue color gradient.

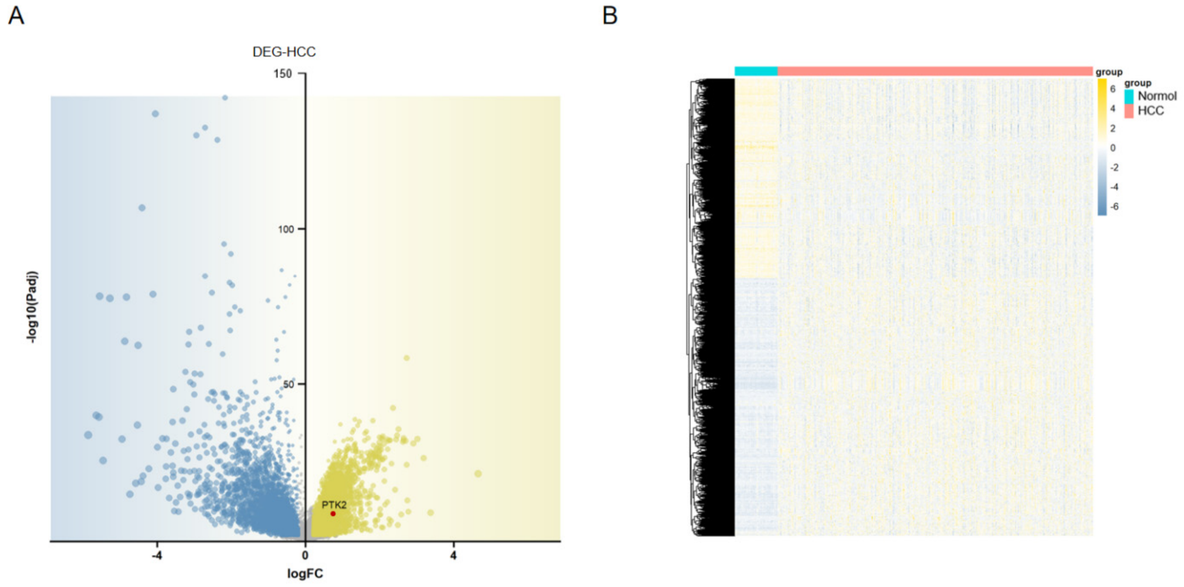

Figure S3. Differential gene expression profiles between HCC and normal liver tissues in the TCGA dataset.

(A) Volcano plot showing the distribution of differentially expressed genes (DEGs), with PTK2 identified as a representative upregulated gene.

(B) Heatmap illustrating hierarchical clustering of DEGs between HCC and normal groups, based on standardized expression levels. Color intensity corresponds to expression level, with red indicating upregulation and blue indicating downregulation.

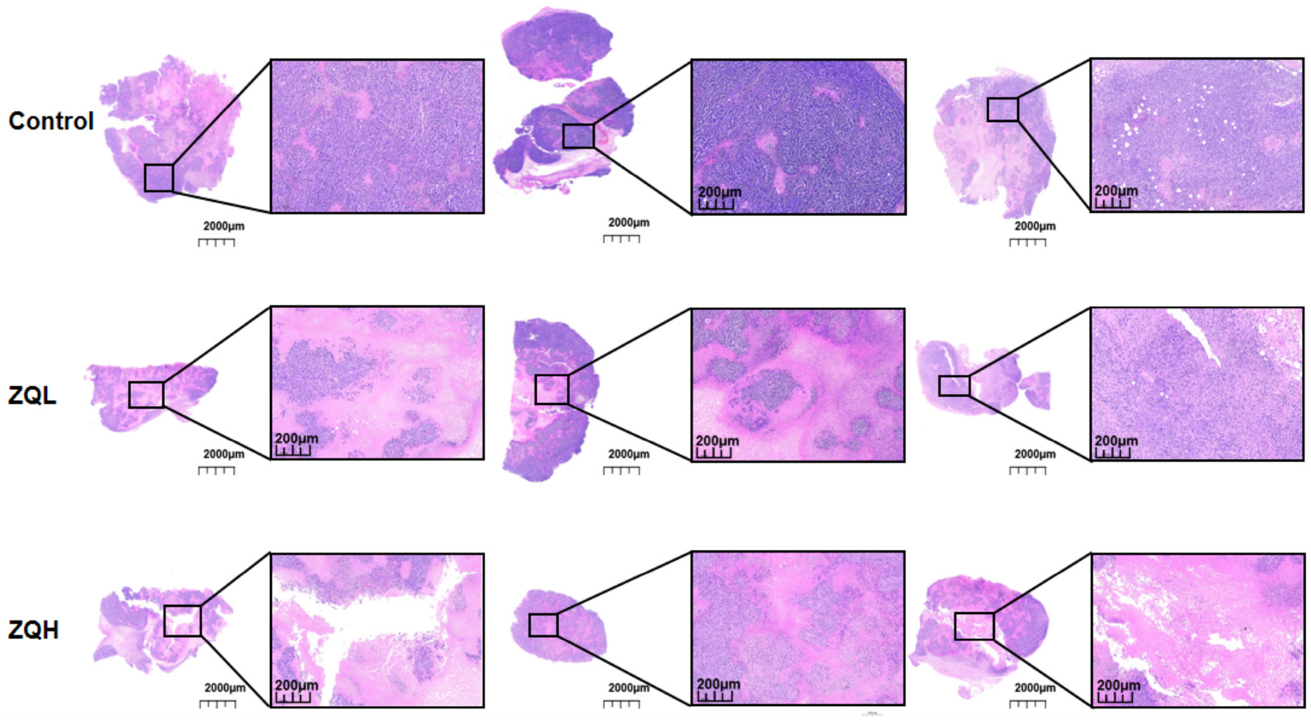

Figure S4. Representative hematoxylin and eosin (H&E) staining of tumor tissues from the control group, low-dose ZQ treatment group, and high-dose ZQ treatment group (n = 3 per group).
Each panel includes low-magnification (scale bar = 2000 μm) and high-magnification views (scale bar = 200 μm) highlighting histopathological alterations among groups.

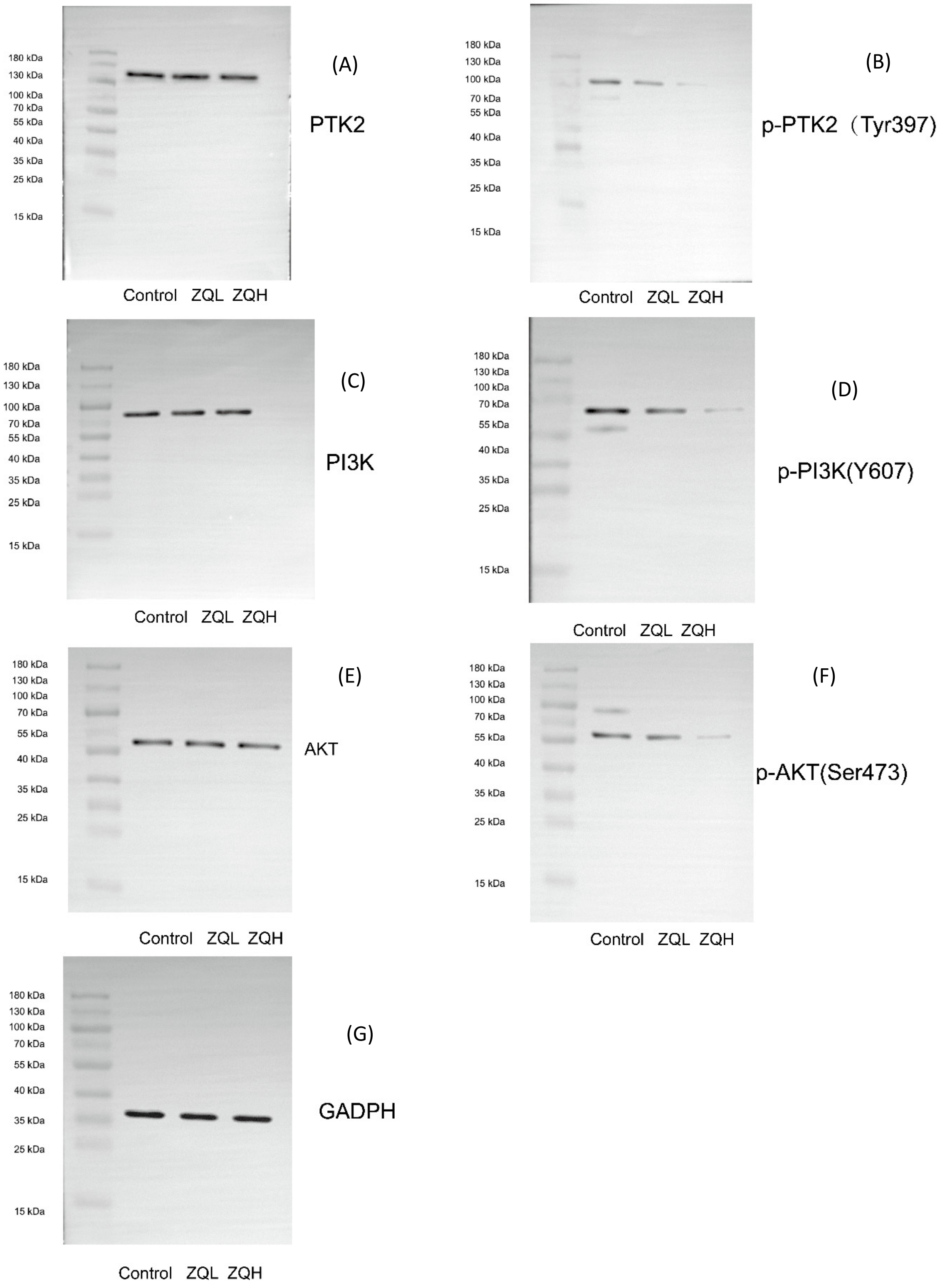

Figure S5. Uncropped and unprocessed full-length Western blot images corresponding to the results

shown in Figure 9A of the main manuscript. A: total PTK2 ;B: p-PTK2 (Tyr397) ; C: total PI3K; D: p-PI3K (Y607) ; E: total AKT; F: p-AKT (Ser473) ; G: GAPDH (loading control). Images are presented in their original format without contrast or brightness adjustment, in accordance with journal guidelines. Lane order (from left to right): Control, ZQL (0.9 g/kg), and ZQH (3.6 g/kg).
